# Engineering Neural Interface Materials for Brain–Computer Interfaces: Toward Reliable Neural Information and Functional Recovery

**DOI:** 10.34133/bmr.0437

**Published:** 2026-11-03

**Authors:** Kaixin Xu, Qinglei Li, Yangmei Dong, Mian Zhong, Weiquan Shi, Bin Lu, Zhiqi Mo, Jaeyoung Park, Jie Wu

**Affiliations:** ^1^ College of Art and Physical Education, Kyungil University, 50, Gamasil-gil, Hayang-eup, Gyeongsan-si, Gyeongbuk-do 38428, Korea.; ^2^ School of Management, Zhengzhou University, Zhengzhou, Henan 475001, PR China.; ^3^ College of Aviation Electronic and Electrical Engineering, Civil Aviation Flight University of China, Deyang 618307, PR China.; ^4^ College of General Education, Zhejiang Vocational College of Special Education, Zhejiang, Hangzhou 310018, PR China.; ^5^ School of Wushu, Henan University, Kaifeng, Henan 475001, PR China.

## Abstract

Motor dysfunction is a major disabling outcome of neurological disorders, including stroke, spinal cord injury, and cerebral palsy. By establishing direct communication between neural activity and external effectors, brain–computer interfaces (BCIs) offer a promising strategy for neural functional reconstruction, enabling motor intention decoding, assistive control, and rehabilitation feedback. However, sustained BCI performance depends not only on advances in decoding algorithms but also on neural interface materials and interfacial properties, as well as the reliable transfer of neural information across signal acquisition, intelligent decoding, control execution, and closed-loop feedback. Existing reviews have mainly focused on invasiveness, electrode technologies, or decoding methods, with comparatively limited integration of neural interface materials, signal reliability, artificial intelligence (AI) adaptability, and closed-loop performance within a unified information reliability framework. Therefore, this review summarizes recent advances in AI-enabled BCIs for motor rehabilitation, compares neural signal and interface characteristics across different levels of invasiveness, examines the effects of key material and interfacial properties on stable signal acquisition, and discusses the roles of AI in data-efficient learning, spatiotemporal and relational neural representation, adaptation to neural distribution shifts, and efficient decoding for real-time closed-loop operation. Representative applications in exoskeletons, neuroprostheses, cerebral palsy rehabilitation, and rehabilitation robotics are further summarized. Finally, current challenges and potential solutions spanning neural interface stability to functional recovery are discussed, providing a systematic pathway toward long-term reliable BCIs and the clinical translation of next-generation intelligent neurorehabilitation systems.

## Introduction

Motor dysfunction is a major disabling outcome of neurological disorders, including stroke, spinal cord injury (SCI), and cerebral palsy (CP), resulting in persistent deficits in voluntary motor control, reduced quality of life, impaired social participation, and substantial long-term healthcare burden [1–4]. Conventional motor rehabilitation predominantly depends on preserved peripheral neuromuscular pathways and facilitates recovery through repetitive task-oriented training, sensory stimulation, and compensatory mechanisms. However, when central motor pathways are structurally damaged or functionally disconnected, motor intentions may remain encoded within the cerebral cortex and related motor networks, while their transmission to peripheral effectors is interrupted. This dissociation creates a critical gap between motor intention generation and motor execution [5–7]. Consequently, re-establishing effective communication between central motor commands and external execution systems while bypassing damaged neural pathways has become a key challenge in neurorehabilitation. Brain–computer interfaces (BCIs), by directly acquiring, decoding, and translating neural signals, offer a promising neurorestorative approach for motor intention decoding, assistive control, sensory feedback, and rehabilitation training, thereby providing new opportunities for functional recovery following neurological injury [8–11].

Since Jacques Vidal introduced the concept of the BCI in 1973 [12], the field has evolved from early proof-of-concept studies into a multilevel neural interface framework encompassing noninvasive scalp electroencephalography (EEG) [8], semi-invasive electrocorticography (ECoG) [13], and invasive intracortical or deep-brain recordings [14,15]. These recording modalities involve distinct trade-offs among spatial resolution, signal-to-noise ratio (SNR), long-term stability, and clinical risk. Noninvasive BCIs are safe, repeatable, and readily deployable, but are susceptible to volume conduction, motion artifacts, and environmental noise [16]. Invasive microelectrodes provide access to high-resolution neural activity and have been used for robotic-arm and cursor control as well as fine-grained decoding of motor intentions [17,18]; however, chronic implantation remains constrained by immune responses, interfacial micromotion, impedance drift, and device longevity. ECoG offers an intermediate balance between signal quality and invasiveness, although chronic tissue responses and long-term signal degradation remain important limitations. Accordingly, the long-term performance of BCIs depends not only on the capacity to acquire neural information but also on the sustained stability of the neural interface.

Recent advances in neural interface materials and structural design, together with AI-based decoding technologies, have provided new opportunities to improve the long-term reliability of BCIs. By optimizing biocompatibility, electrical conductivity and electrochemical properties, interfacial adhesion, and mechanical compliance, neural interface materials can improve material–tissue coupling and mitigate interface instability, thereby providing a foundation for stable long-term neural signal acquisition [19–23]. In parallel, AI models can enhance neural decoding under nonstationary conditions and interindividual variability through feature learning, temporal modeling, cross-subject transfer, and adaptive updating [8,9,24–26]. Reliable decoding can subsequently drive external effectors, including exoskeletons [27], neuroprostheses [8], and rehabilitation robots [28], while integration with multimodal feedback enables closed-loop training that may support neuroplastic modulation and motor functional recovery. However, existing BCI reviews have largely addressed levels of invasiveness, electrode materials, or decoding algorithms as separate topics, with comparatively limited attention to the continuous transmission of neural information from interface-level acquisition to functional recovery and the progressive loss of reliability along this pathway. In motor rehabilitation BCIs, the reliability of individual stages is not independent. Upstream changes in material–tissue coupling, interfacial impedance drift, tissue responses, and channel failure can lead to signal degradation, which may manifest downstream as feature drift and decoding uncertainty, subsequently resulting in control errors and feedback mismatch and ultimately limiting rehabilitation benefits [29,30]. Thus, the long-term reliability of a BCI cannot be defined by a single material property, a single-session decoding accuracy, or a particular type of end device; rather, it depends on whether neural information can be transmitted continuously and reliably across successive stages of the system. From this perspective, neural interface and information acquisition, neural signal reliability, neural representation and AI-based decoding, control execution, closed-loop feedback, and neuroplasticity and functional recovery can be integrated into an “information reliability chain” that captures both information transmission and failure propagation throughout motor rehabilitation BCIs (Fig. 1).

**Fig. 1.**
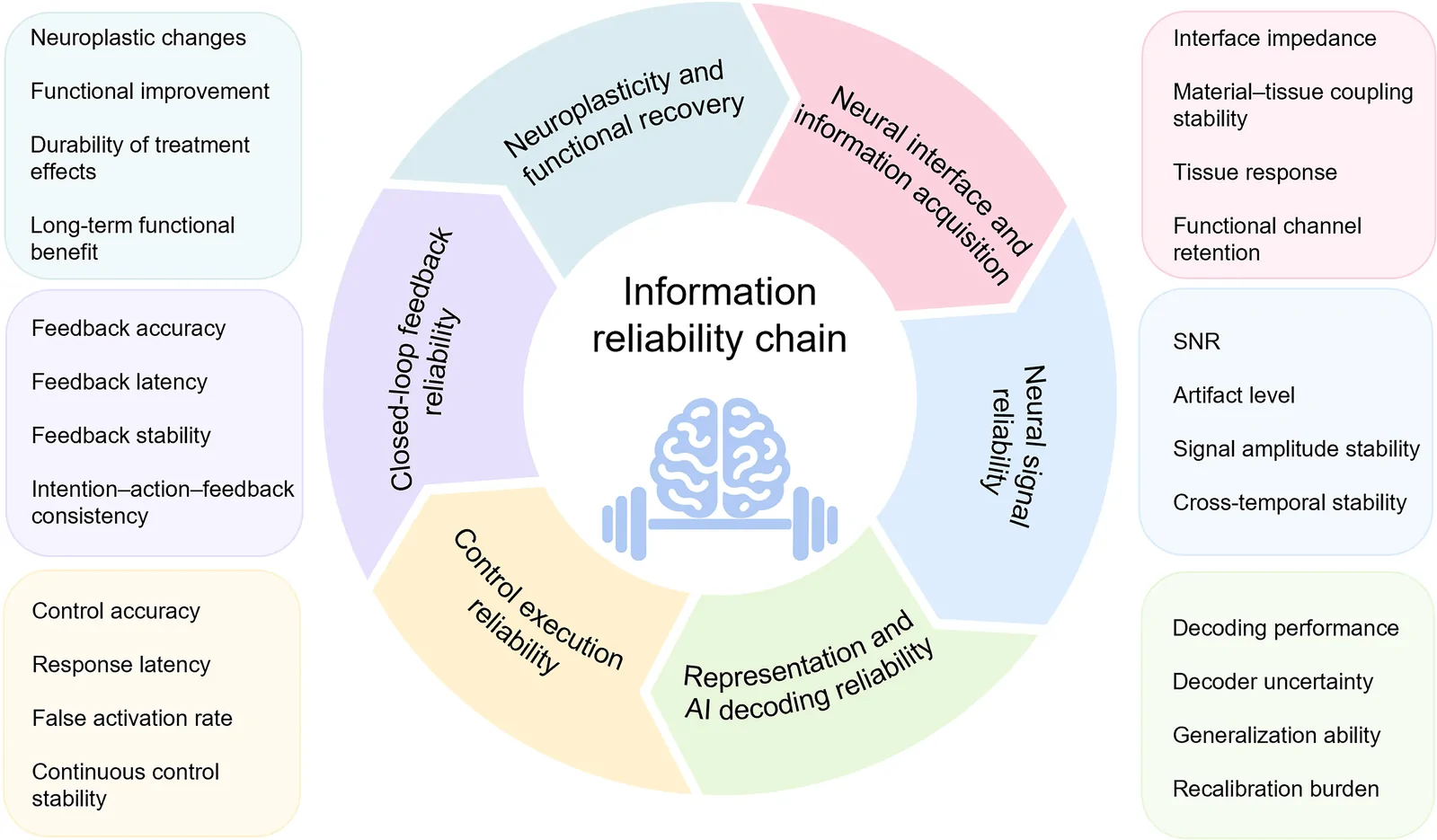
Information reliability chain and key evaluation metrics for motor rehabilitation BCIs, encompassing neural interface and information acquisition, neural signal reliability, representation and AI decoding reliability, control execution reliability, closed-loop feedback reliability, and neuroplasticity and functional recovery.

Structured around this information reliability chain, this review follows a central theme of “from neural interfaces to functional recovery” and systematically examines advances in neural interface materials, AI-based decoding, and BCI-enabled motor rehabilitation. First, noninvasive, semi-invasive, and invasive BCIs are compared in terms of their signal sources, interface characteristics, and boundaries for long-term application, thereby clarifying the advantages and limitations of different neural recording modalities at the information acquisition stage. Second, the effects of biocompatibility, electrical conductivity and electrochemical properties, interfacial adhesion, and mechanical matching and fatigue reliability on stable long-term signal acquisition are examined from the perspective of material and interface design. The roles of AI in addressing key decoding reliability challenges are then discussed, including data-efficient learning with limited neural data, spatiotemporal and relational neural representation, generalization and adaptation under neural distribution shifts, and efficient decoding for real-time closed-loop operation. The review further examines how reliable neural decoding can be translated into motor assistance through external effectors, including exoskeletons, neuroprostheses, and rehabilitation robots, and how closed-loop feedback can further support motor rehabilitation and functional recovery. Finally, key challenges related to long-term interface stability, model generalization, control and feedback reliability, and clinical evidence are critically assessed, and future directions for BCI-enabled motor rehabilitation are discussed from an integrated information reliability chain perspective, providing a framework for the neural interface design, functional recovery, and clinical translation of long-term reliable BCIs.

## Classification of BCIs

In motor rehabilitation BCIs, the information reliability chain begins with the stable acquisition of neural information, the quality of which directly affects subsequent AI-based decoding, closed-loop feedback, and functional recovery. Different neural recording modalities establish distinct spatial relationships between electrodes and neural tissue, resulting in different trade-offs among signal sources, spatial resolution, SNR, interface stability, and long-term applicability [31]. As BCIs have evolved from early signal decoding toward high-density recording, multimodal integration, and closed-loop modulation, the research focus has shifted from short-term signal acquisition and device control to the continuous and reliable transmission of neural information during long-term rehabilitation [32].

Based on the implantation location of the neural recording interface and its spatial relationship with the brain parenchyma, neural recording modalities in BCIs are commonly classified as noninvasive, semi-invasive, and invasive, and this 3-level classification is adopted in this review. Noninvasive interfaces are located outside the cranium, semi-invasive interfaces are positioned intracranially without penetrating the brain parenchyma, whereas invasive interfaces extend into the brain tissue [33]. This classification primarily reflects the anatomical location of the interface and the extent of tissue penetration and does not fully correspond to the degree of surgical invasiveness. For example, although subdural ECoG does not penetrate the brain parenchyma, it generally requires more extensive cranial exposure; in contrast, stereo-electroencephalography (sEEG) penetrates the brain parenchyma but can be implanted along stereotactic trajectories through relatively small cranial openings [34]. Therefore, the actual degree of invasiveness may also depend on the position relative to the dura mater, tissue penetration, surgical approach, and recording depth. Within this framework, this section outlines key developments in BCI and compares the signal acquisition characteristics, interface conditions, and long-term application boundaries of different neural recording modalities, including their material and interface characteristics, spatial sampling characteristics, temporal acquisition parameters, tissue responses, and long-term recording performance (Table 1).

**Table 1.** Material and interface characteristics and long-term recording performance of different BCI neural recording modalities

BCI type	Material system	Neural recording site	Spatial/temporal resolution	Tissue response	Long-term recording performance	Refs.
Noninvasive (EEG)	SA/PAM–KCl–PEDOT:PSS double-network hydrogel	Scalp EEG (O1/P3; F7/F8/Fp1/Fp2)	Temporal: sampling rate, 1,000 Hz	No cytotoxicity; no skin irritation/inflammation after 7 d; no skin damage after 12 h	Stable EEG/PSD recording for 12 h; contact impedance of 0.4 kΩ maintained for 12 h; ERP/PSD preserved after 14 d of repeated use	[58]
Noninvasive (EEG)	PEDOT:PSS/PAMPS–LiCl–glycerol hydrogel (POLiTAG)	Motor cortex (C4); prefrontal cortex (FCz)	NR	No skin irritation or visible marks after 6 h of continuous wear	Low interfacial impedance maintained for 29 d; scalp impedance remained <30 kΩ for 4 weeks; stable detection of motor-imagery SMR and ErrP	[198]
Noninvasive (fNIRS)	Polyimide fPCB + LED/photodetector	Bilateral primary motor cortex (M1; left C1–C3, right C2–C4)	Spatial: 3D optode localization error, 4.2 mm; temporal: single-channel acquisition, 7.5 ms; module frame rate, 11 Hz	NR	Flexible module improved scalp conformity; 18 g/module; wearable measurements supported; real-time optode-position tracking during movement	[62]
Noninvasive (fNIRS)	Dual-wavelength LED/SiPM + PDMS optical coupling + Parylene C encapsulation	Left prefrontal cortex (PFC)	Spatial: source–detector distances, 15.2/17.7/ 20.2 mm; temporal: 10 Hz	NR	PDMS interface optimized optical coupling, increasing signal intensity by up to 22×; wireless BLE transmission; continuous operation for 50 h; real-time monitoring of HbO/HbR changes	[199]
Noninvasive (OPM-MEG)	Biaxial/triaxial optically pumped magnetometer (OPM) array	Bilateral sensorimotor cortex	Spatial: 10 mm; temporal: sampling rate, 1,500–6,000 Hz; bandwidth, 0–150 Hz	NR	Stable detection of sensorimotor β modulation; reproducible localization across 30 trials	[65]
Noninvasive (OPM-MEG)	80-channel single-axis OPM whole-head array	Left sensorimotor cortex	Spatial: 5-mm source-space grid; temporal: sampling rate, 1,000 Hz	NR	High consistency across repeated resting-state recordings; microstate spatial correlations of 0.74–0.98, with most semi-partial correlations >0.6	[200]
Semi-invasive (μECoG, epidural)	Flexible PI array + Pt/IrOx/PEDOT:PSS electrodes	Primary somatosensory cortex (S1BF)	Spatial: electrode diameter, 10/100 μm; spacing, 45 μm; temporal: sampling rate, 12,207 Hz	8-μm conformal array caused no apparent cortical compression or neural damage; chronic implantation induced mild inflammation	Stable single-unit recordings for up to 12 weeks; IrOx/PEDOT:PSS electrodes could still distinguish stimulus-evoked spikes at 12 weeks	[69]
Semi-invasive (ECoG, epidural)	WIMAGINE dual 64-electrode arrays + hermetically sealed implant	Bilateral sensorimotor cortex	Spatial: electrode spacing, 4 × 4.5 mm	One subcutaneous implant was explanted due to infection; the other implant showed no infection and remained functional	Stable ECoG recording for nearly 1 year; power attenuation, 0.03 dB/day; arm-decoding accuracy maintained at 0.97 ± 0.1 for 11 months	[48]
Semi-invasive (subdural μECoG)	PVA/PTPM hydrogel + flexible PI/ PEDOT:PSS array	Left visual cortex; somatosensory cortex	Spatial: submillimeter scale	Markedly reduced GFAP/Iba1-associated glial responses and fibrotic encapsulation; no appreciable change in neuronal density	Stable SSVEP recording for 16 weeks; SNR retention of 94.8%; stable long-term impedance	[113]
Semi-invasive (subdural μECoG)	TiN microelectrodes + flexible CMOS chip	Somatosensory cortex, M1/S1; visual cortex (V1/V2/V4)	Spatial: electrode pitch, 26.5 × 29 μm; temporal: sampling rate, 33.9/ 8.475 kS/s	No marked pathological changes beneath the electrodes; only mild glial cell proliferation; slight mechanical damage at the implantation margins	Stable recordings for up to 64 d; stable visually evoked localization from day 36 to day 62	[201]
Invasive (sEEG)	Flexible PI/PtNR depth electrodes	Barrel cortex, somatosensory cortex, visual cortex, and left lateral geniculate	Spatial: electrode spacing, 60 μm	GFAP-positive area at 14 d post-implantation was lower than that with clinical sEEG; no appreciable reduction in neuronal density	Chronic recording for 25 d with preserved cross-day LFP/HGP responses; no appreciable degradation after accelerated aging for 150 d and 84,000 bending cycles	[202]
Invasive (sEEG)	Pt multi-contact depth electrodes	Primary sensorimotor, supplementary motor, and premotor cortices	Spatial: center-to-center contact spacing, 3.5 mm; temporal: 1,200 Hz	NR	Reliable localization of high-frequency activity associated with hand, foot, and tongue movements; 99% agreement between sensorimotor functional localization and stimulation results	[79]

### Historical evolution of BCIs

The development of BCIs has consistently revolved around 2 fundamental questions: whether neural activity can be acquired in a stable and reliable manner, and whether such activity can be decoded and transformed into information transfer, external control, or rehabilitation feedback [35]. From this perspective, the history of BCI development should not be viewed merely as a sequence of technological milestones but rather as a continuous process of improving neural information reliability—from making neural activity measurable to enabling its decoding, modulation, closed-loop integration, and ultimately long-term application. Early EEG studies established the foundation for noninvasive neural signal acquisition. Through the systematic recording and characterization of human electroencephalographic activity, Berger [36] transformed brain electrical signals into quantifiable experimental observations and laid the methodological foundation for subsequent scalp-based neural acquisition in BCI systems. To further improve recording depth, spatial specificity, and long-term signal accessibility, research subsequently expanded toward invasive neural recording strategies. The transcutaneous electrode and cannula implantation approach proposed and implemented by Lilly [37] provided an early engineering prototype for chronic intracranial recording and electrical stimulation studies. Subsequently, Fetz [38] demonstrated operant modulation of single-neuron firing activity through closed-loop animal experiments, providing the first evidence that neural activity can not only be recorded but also trained and regulated under feedback reinforcement. This work established an important experimental basis for the closed-loop logic that later became central to BCI systems, linking neural activity, external feedback, and behavioral adaptation. Around the same period, Vidal [12] formally introduced the concept of the BCI and incorporated neural signal decoding and external device control into a unified engineering framework, marking the transition of BCI research from neural recording toward conceptual establishment and prototype validation. Within this emerging framework, invasive approaches were among the first to demonstrate the feasibility of chronic intracortical interfacing. Kennedy [39] developed a cone electrode that promoted neurite ingrowth following implantation into the rat cortex, enabling stable, long-term recording of single- and multi-unit neural activity. Farwell and Donchin [40] established one of the earliest classical BCI paradigms by enabling character selection through P300 event-related potentials, demonstrating attention-driven information interaction and laying the foundation for subsequent communication-oriented BCI systems.

Since the beginning of the 21st century, BCIs have evolved from simple task-oriented control systems toward platforms capable of decoding complex behavioral representations and supporting clinical translation, accompanied by major advances in both neural decoding capability and system-level implementation. Based on population-level cortical neuronal activity, Carmena et al. [41] achieved real-time closed-loop control of a robotic arm, demonstrating that continuous motor decoding can be stably maintained under online feedback conditions. As demands for higher control dimensionality and more complex decoding increased, semi-invasive interfaces and high-dimensional neural recording further expanded the information capacity of BCI systems. Leuthardt et al. [42] showed that ECoG offers superior spatiotemporal resolution, broader frequency coverage, and greater clinical feasibility, facilitating its adoption in motor- and language-related BCI applications. Building on high-frequency cortical activity, subsequent studies achieved effective decoding of speech-related neural representations in humans [43], demonstrating the ability of BCIs to extract increasingly complex neural information beyond conventional motor functions. Meanwhile, advances in neural decoding and multidimensional signal integration enabled BCIs to evolve from low-dimensional command generation to coordinated motor control. Ifft et al. [44] realized real-time decoding of bilateral arm kinematics, enabling nonhuman primates to synchronously control virtual bimanual movements using neural activity alone. This study represented a key step toward multi-degree-of-freedom control and highlighted the growing capacity of BCIs to convert richer neural information into complex behavioral outputs.

In recent years, BCI development has increasingly moved toward clinical translation and long-term therapeutic application. Ajiboye et al. [45] used continuous decoding of invasive cortical signals to control multichannel functional electrical stimulation, restoring reaching and grasping abilities in individuals with paralysis. This work marked a shift from external device control to reconstruction of impaired neuromotor pathways. Meanwhile, artificial intelligence (AI) has become a key bridge between neural activity and functional output. Moses et al. [46] applied AI-based modeling of high-gamma ECoG signals to decode language-related information during natural tasks, demonstrating the growing ability of intelligent decoding to transform complex neural dynamics into meaningful behavioral outcomes. As neural information flow evolved from one-way control to bidirectional interaction, closed-loop sensory restoration emerged as a major direction in BCI development. Flesher et al. [47] generated stable and distinguishable tactile sensations through direct cortical stimulation, laying the foundation for sensorimotor closed-loop BCIs and highlighting the importance of feedback in functional recovery. Meanwhile, advances in wireless and closed-loop technologies have enabled the long-term stable operation and clinical application of implantable BCIs. Lorach et al. [48] developed a brain–spine interface that restored natural walking in individuals with SCI through real-time decoding of cortical motor intentions and modulation of spinal cord stimulation. Similarly, the BCI NEO system advanced wireless implantation and real-time neural transmission, indicating the transition of BCIs from laboratory prototypes to clinically sustainable neurotechnologies [49]. Noninvasive BCIs have also continued to overcome limitations in signal acquisition stability and fine motor control. Ding et al. [8] achieved real-time single-finger robotic control using scalp EEG, demonstrating the potential of noninvasive neural signals for fine motor applications. Dutta et al. [50] developed a thermoresponsive ionic biogel neural interface that enabled long-term stable EEG acquisition across hair and monitoring of tactile neural responses. More recently, Chandrasekaran et al. [51] developed a dual neural bypass system that integrated a bidirectional intracranial BCI with brain–spinal neuromodulation, enabling voluntary hand movement, tactile feedback, and sustained sensorimotor functional recovery in an individual with complete tetraplegia. This study reflects the progression of BCIs from immediate functional assistance toward sensorimotor closed-loop operation and sustained neurorehabilitation. Overall, BCI evolution reflects a continuous progression from neural recording to reliable decoding, from unidirectional control to bidirectional closed-loop interaction, and from short-term demonstrations to long-term clinical translation (Fig. 2) [8,12,36,37,40,41,43–48,50,51]. For motor rehabilitation, future advances will rely not only on improving decoding performance but also on establishing an information reliability chain encompassing neural interfacing, signal stability, intelligent decoding, and rehabilitation feedback.

**Fig. 2.**
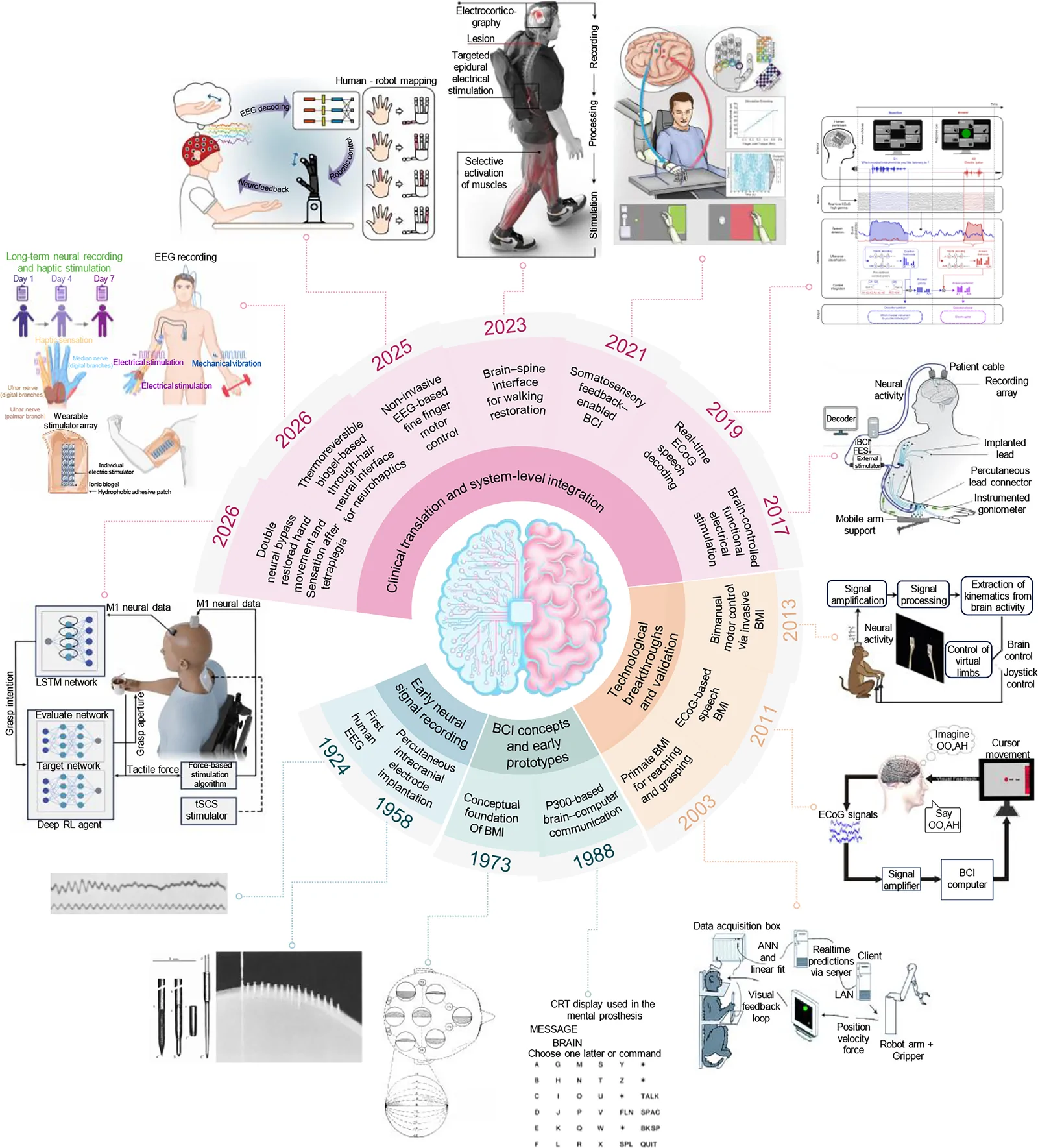
Historical development of BCIs. Reproduced from Ref. [36]. Public domain. Reproduced from Ref. [37] with permission from AAAS. Reproduced from Ref. [12] with permission from Annual Reviews. Reproduced from Ref. [40] with permission from Elsevier. Reproduced from Ref. [41] under the Public Library of Science Open-Access License. Copyright © 2003 Carmena et al. Reproduced from Ref. [43]. © IOP Publishing. Reproduced with permission. All rights reserved. Reproduced from Ref. [44] with permission from AAAS. Reproduced from Ref. [45] with permission from Elsevier. Reproduced from Ref. [46] under the CC BY 4.0 license. Copyright © 2019 The Author(s). Reproduced from Ref. [47] with permission from AAAS. Reproduced from Ref. [48] under the CC BY 4.0 license. Copyright © 2023 The Author(s). Reproduced from Ref. [8] under the CC BY 4.0 license. Copyright © 2025 The Author(s). Reproduced from Ref. [50] under the CC BY-NC 4.0 license. Copyright © 2026 The Authors. Reproduced from Ref. [51] under the CC BY 4.0 license. Copyright © 2026 The Author(s).

### Noninvasive BCIs

Within the information reliability chain, noninvasive BCIs offer low risk, repeatable deployment, and high adaptability to real-world settings. However, neural information is acquired indirectly by external sensors after passing through the brain tissue, skull, and scalp, making the recorded signals more susceptible to volume conduction, superficial physiological activity, and environmental interference [16]. Current noninvasive BCI modalities mainly include EEG [52], functional near-infrared spectroscopy (fNIRS) [53], and magnetoencephalography (MEG) [54], which characterize brain function from different physiological dimensions, including electrical activity, hemodynamic responses, and magnetic fields induced by neuronal currents. Among these modalities, EEG records potential fluctuations generated by synchronized activity of large populations of cortical neurons through scalp electrodes [55]. Specifically, postsynaptic potentials of cortical pyramidal neurons generate equivalent dipole fields within brain tissue, which propagate to the scalp through volume conduction and are subsequently detected by surface electrodes (Fig. S1A) [56]. Therefore, in addition to skull attenuation and volume conduction, the electrical and mechanical stability of the electrode–skin interface is a key determinant of EEG signal fidelity. Although conventional wet electrodes can reduce interfacial impedance through conductive gels, gel dehydration and interfacial changes can readily induce impedance drift during long-term recording [57]. In contrast, dry, semi-dry, flexible, and hydrogel electrodes can improve scalp contact and reduce motion artifacts by optimizing electrical conductivity, mechanical compliance, and interface stability, thereby providing a material basis for stable long-term EEG recording. For example, Xue et al. [58] developed a conductive–adhesive bilayer semi-dry hydrogel electrode, in which KCl and poly(3,4-ethylenedioxythiophene) (PEDOT): poly(styrenesulfonate) (PSS) enhanced ionic/electronic conduction, while glycerol-mediated water retention and an adhesive layer stabilized the interface. The electrode exhibited a skin-contact impedance below 0.4 kΩ and remained stable during 12 h of continuous monitoring, whereas conventional wet electrodes failed after 5 h due to drying of the conductive paste. Moreover, the electrode enabled stable acquisition of N170 and P300 event-related potentials, demonstrating that a stable electrode–skin interface is essential for maintaining long-term EEG signal quality.

fNIRS measures task-related changes in oxyhemoglobin and deoxyhemoglobin concentrations through variations in near-infrared light absorption, thereby reflecting local perfusion and oxygen regulation associated with neurovascular coupling [59]. Compared with EEG, fNIRS is less sensitive to electromagnetic interference and is therefore more suitable for naturalistic scenarios, pediatric populations, and mild motor tasks. However, its hemodynamic response exhibits an inherent delay on the order of seconds, and signals acquired under conventional source–detector separation often contain contributions from both cortical activity and superficial extracranial blood flow. To improve cortical signal specificity, short-separation channels and short-channel regression are commonly employed to suppress contamination from systemic superficial hemodynamics (Fig. S1B) [60]. In addition to physiological interference, interfacial coupling between the light sources/detectors and the scalp is an important factor affecting fNIRS signal reliability. Hair obstruction, probe pressure, and motion-induced relative displacement can alter optical coupling and the effective optical path length, thereby increasing signal fluctuations and motion artifacts [61]. Accordingly, recent wearable fNIRS systems have increasingly incorporated flexible substrates, miniaturized light sources and detectors, and conformal probe designs to improve head conformity and stability during movement. For example, Xu et al. [62] developed a modular fNIRS system based on flexible printed circuit boards, in which lightweight and bendable probes enhanced conformal coupling at the optode–scalp interface. The mean radial distances of the rigid and flexible probes on a head phantom were 107.0 ± 3.7 mm and 104.8 ± 2.8 mm, respectively, with the latter being closer to the ideal fitting distance of 105 mm. Meanwhile, the SNR of the intra-module channels reached 65 to 72 dB. This flexible architecture improved probe conformity to the curved surface of the head, providing a structural basis for stable fNIRS optical signal acquisition.

MEG records weak magnetic fields generated outside the head by synchronized postsynaptic currents of cortical pyramidal neurons (Fig. S1C) [63]. Early studies by Hämäläinen et al. [64] demonstrated that MEG can theoretically provide superior source localization compared with EEG while maintaining millisecond-level temporal resolution. However, conventional MEG based on superconducting quantum interference devices relies on cryogenic systems and magnetically shielded environments, limiting its deployment in real-world motor rehabilitation settings. In recent years, wearable MEG based on optically pumped magnetometer (OPM) has provided a new approach to overcome these limitations. Spedden et al. [65] demonstrated the feasibility of OPM-MEG for stable magnetic signal recording during natural gait and stepping tasks, promoting the transition of MEG from fixed laboratory equipment toward mobile platforms for brain function recording. Overall, noninvasive BCIs offer advantages in safety and deployability and therefore represent the most broadly applicable neural information entry point in motor rehabilitation. However, their signal reliability remains jointly constrained by spatial resolution, artifact interference, hemodynamic delay, and environmental noise. Therefore, subsequent AI-based decoding and closed-loop feedback in noninvasive BCIs rely more heavily on high-quality preprocessing, multimodal integration, and adaptive models to compensate for uncertainty in upstream neural information.

### Semi-invasive BCIs

Semi-invasive BCIs are primarily represented by ECoG, in which electrode arrays are typically placed either on the subdural cortical surface or epidurally for recording across the dura, enabling acquisition of local field potentials (LFPs) and broadband high-gamma activity without penetrating brain parenchyma [66]. Within the information reliability chain, ECoG occupies an intermediate position between noninvasive EEG and intracortical microelectrodes, combining relatively high SNR, broad frequency coverage, and lower risk of tissue injury. As such, it is regarded as an important compromise solution for high-dimensional continuous decoding and long-term translation of neural interfaces. In particular, the position of the electrode relative to the dura directly alters the physical barrier between the neural source and the sensing interface, resulting in a trade-off between signal quality and invasiveness [67]. The quality of ECoG signals is closely associated with electrode placement, dural barriers, array density, and recording frequency bands. Branco et al. [68] compared epidural and subdural recordings and found that epidural signals generally exhibit lower amplitudes because of attenuation and filtering effects introduced by the dura, with these differences becoming more pronounced in high-density arrays and higher-frequency bands. Nevertheless, from a decoding perspective, epidural approaches can still preserve usable neural information in certain tasks and may reduce surgery-related risks (Fig. S1D). In addition to implantation location, the material and structural characteristics of ECoG arrays also influence the long-term stability of the electrode–cortex interface. Vomero et al. [69] developed a polyimide (PI)-based μECoG array and demonstrated that device conformability depends not only on material modulus but also on substrate thickness and geometry. An 8-μm-thick PI device formed a stable conformal interface with the brain surface, whereas a nonconformal 12-μm-thick device exhibited a marked increase in impedance and cortical indentation after 3 weeks of implantation. The conformal device further enabled high-frequency neural activity recording for up to 12 weeks, indicating that regulating overall mechanical compliance through substrate thickness and geometry can reduce interface instability and tissue deformation, thereby maintaining long-term ECoG information reliability.

Building on a mechanically stable interface, ECoG arrays also require electrode miniaturization and high-density layouts to improve the spatial sampling of local neural activity. As summarized by Moon et al. [70], μECoG technology employs microfabricated high-density arrays to overcome the resolution limitations of conventional macroscale ECoG while maintaining broad cortical coverage, thereby providing greater information capacity for fine functional mapping and high-dimensional neural decoding (Fig. S1E). This spatial sampling capability is particularly important for capturing high-frequency neural activity with local cortical specificity. High-gamma activity in ECoG is closely associated with local neuronal population activity and represents an important information source for motor, sensory, and speech BCIs [71,72]. Accordingly, the fine spatial information acquired by high-density arrays can be further integrated with task-related neural feature modeling, enabling the transition from stable signal acquisition to functional information decoding. Metzger et al. [73] further combined high-density μECoG arrays with nonlinear decoding models to characterize individualized high-gamma features, substantially improving decoding performance in a speech neuroprosthesis. Building on this approach, dynamic mapping of language-related cortical activity using high-density ECoG arrays combined with imaging-based localization has also provided an important basis for functional localization and model training in speech BCIs (Fig. S1F) [74]. However, high spatial resolution and favorable decoding performance do not necessarily ensure stable long-term information input from ECoG. In a chronic subdural ECoG study, Yan et al. [75] observed that signal amplitude could decrease over time during long-term implantation, indicating that tissue responses, electrode encapsulation, wireless transmission, and interface stability can still affect long-term signal quality. Therefore, ECoG interface design should not focus solely on increasing array density and short-term signal quality. Material flexibility, structural conformability, interfacial impedance, and chronic tissue responses should also be considered to balance spatial sampling capability with long-term interface stability, thereby maintaining reliable neural information input during long-term BCI applications.

### Invasive BCIs

Invasive BCIs acquire neural activity by implanting electrodes directly into the brain parenchyma or intracortical regions, providing high spatial resolution and information capacity [76]. The close proximity of the electrodes to neural sources enables the recording of LFPs, high-frequency neuronal population activity, and even single-neuron spiking, providing rich input for high-dimensional neural decoding. sEEG is an important form of invasive deep-brain recording, in which stereotactically guided depth electrodes are inserted into the brain parenchyma along 3-dimensional trajectories, enabling spatially selective sampling of cortical sulci and gyri as well as subcortical structures without extensive craniotomy [77]. Iida and Otsubo [78] demonstrated 3-dimensional coverage of complex cortical folds and subcortical structures using depth electrodes implanted along multiple trajectories (Fig. S1G). Jensen et al. [79] further recorded task-related high-frequency neural activity using multi-contact sEEG electrodes to localize motor- and language-related functional regions (Fig. S1H). However, the advantages of sEEG are primarily associated with deep-network sampling and clinical functional localization, whereas limitations remain in high-density sampling of local neural activity and long-term continuous recording. In contrast, intracortical microelectrode systems directly implant microelectrode arrays into the motor cortex or other functional regions to record single-neuron action potentials, multi-unit activity, and LFPs, representing an important approach for high-spatial-resolution neural information acquisition. Representative configurations include microwire electrode arrays [80], Utah arrays [29], Michigan arrays [81], and flexible high-channel-count neural probes [82]. For example, Zhao et al. [83] developed a high-density intracortical array using ultraflexible nanoelectronic thread electrodes, achieving a neural recording density of approximately 1,000 neural units mm^−3^ while reducing the dimensions of the implanted structure and maintaining stable recordings for several months.

As recording systems become increasingly miniaturized and densely integrated, the long-term interface stability associated with direct electrode implantation into brain tissue becomes increasingly important. Material–tissue mechanical mismatch, interfacial micromotion, chronic inflammation, and device aging can alter electrode–neural tissue coupling, further leading to impedance drift, signal attenuation, and a reduction in functional recording channels. Accordingly, the development of invasive BCIs has expanded beyond the pursuit of short-term, high-resolution recording toward maintaining chronic neural interface stability through material and structural optimization. For example, Roh et al. [84] introduced soluble poly(vinyl alcohol) (PVA) as a transient supporting material that provided mechanical stiffness before implantation and rapidly dissolved after implantation, reducing the bending stiffness of the flexible mesh electrode by approximately 1,078-fold. This design mitigated the mechanical mismatch between the electrode and brain tissue during long-term implantation and resulted in a low chronic tissue response over 6 weeks. For chronic interface regulation after implantation, Vomero et al. [85] further developed a 12-μm-thick PI-based flexible intracortical electrode and compared probe widths of 30 and 100 μm to investigate the influence of device geometry on the chronic interface. Both devices enabled stable neural recording for up to 12 weeks, while the smaller electrode exhibited a lower surrounding glial fibrillary acidic protein (GFAP) response, indicating that the coordinated regulation of material compliance and device dimensions can improve electrode–tissue interface integration and maintain stable chronic neural recording.

Overall, different BCI neural recording modalities establish distinct spatial relationships between neural sources and sensing interfaces, resulting in different trade-offs among safety, signal quality, information capacity, and long-term stability. Noninvasive interfaces offer higher safety but are more susceptible to signal attenuation, artifacts, and fluctuations in interfacial coupling. ECoG improves SNR and spatial sampling capability but remains limited by long-term interface stability. sEEG and intracortical microelectrodes can acquire deeper or higher-resolution neural information, although tissue invasion and the risk of chronic interface instability are further increased. Thus, the recording modality determines the input characteristics of the information reliability chain, while material and structural design further influence reliable neural information acquisition by regulating interfacial coupling and long-term stability between the electrode and the skin or neural tissue. Therefore, after defining the information acquisition boundaries of different recording modalities, further attention should be directed toward the materials and key properties of neural interfaces and their effects on signal reliability.

## Key Performance Requirements of Neural Interfaces for BCIs

Within the information reliability chain, neural interfaces not only convert neural activity into engineering signals, but their material properties and interfacial states also directly determine whether neural information can be acquired stably over the long term. Biological, electrical, and mechanical interactions between the material and the skin or neural tissue can further affect signal noise, drift, and long-term recording reliability through tissue responses, interfacial impedance, contact stability, and mechanical micromotion [86]. Therefore, the design of BCI neural interfaces should not focus on a single material property, but rather coordinate biocompatibility, electrical conductivity and electrochemical performance, interfacial adhesion, and mechanical matching and fatigue reliability to maintain stable material–tissue coupling and signal transmission. This section focuses on these 4 key properties, examining the corresponding material design strategies and their effects on the upstream stability of the information reliability chain.

### Biocompatibility

Biocompatibility is the foundation for the long-term reliable operation of BCI neural interfaces. Its essence is not limited to the single criterion of material “nontoxicity”, but rather reflects a dynamic interfacial state jointly determined by materials, biological tissues, and the operating environment [22,87,88]. For invasive BCIs, implantation-induced tissue injury, blood–brain barrier disruption, inflammatory cell recruitment, glial scar formation, and electrode micromotion progressively increase the effective recording distance, leading to elevated interfacial impedance, increased noise, and eventual loss of recording channels [89]. For noninvasive BCIs, prolonged wear introduces challenges associated with the stratum corneum barrier, sweat electrolytes, sebum secretion, variations in temperature and humidity, and mechanical friction, which may induce erythema, itching, contact irritation, or signal drift. Therefore, biocompatibility directly determines whether BCIs can transition from short-term experimental demonstrations to long-term monitoring, home-based rehabilitation, and clinical application. For noninvasive epidermal interfaces, skin tolerance and long-term contact stability remain primary considerations. To address this issue, Wang et al. [90] developed an antibacterial semi-dry hydrogel electrode based on poly(N-acryloyl glycinamide) and hydroxypropyltrimethyl ammonium chloride chitosan (HACC), with antibacterial activity arising from electrostatic interactions between positively charged HACC and bacterial cell membranes. At 3 wt % HACC, the inhibition zone diameters against *Escherichia coli* and *Staphylococcus epidermidis* reached 20.75 ± 1.08 mm and 15.17 ± 0.54 mm, respectively. No obvious skin irritation was observed after up to 7 d of skin contact, and the hydrogel could be repeatedly used for EEG acquisition over 21 d, demonstrating good biocompatibility under prolonged contact and repeated use. However, long-term validation was primarily based on intermittent use, leaving skin tolerance during continuous wear under sweat accumulation, thermal and humidity variations, and interfacial disturbances insufficiently evaluated. To address this limitation, Wang et al. [91] developed a 2.7-μm-thick ultrathin hydrogel electrode using a gelatin matrix reinforced with a polyurethane nanomesh and crosslinked with vanillin. Dynamic Schiff-base crosslinking introduced by vanillin imparted antibacterial activity, with inhibition zone diameters of 8.3 and 13.7 mm against *E. coli* and *Staphylococcus aureus*, respectively, while L-929 cell viability remained at 96.7% after 7 d of culture. The ultrathin structure further facilitated gas and water-vapor transport and withstood 1,000 stretching cycles at 100% strain. During 8 d of continuous wear, the skin-contact impedance remained below 55 kΩ without obvious skin irritation or discomfort, demonstrating interface stability and biocompatibility under prolonged wear and dynamic deformation. Beyond hydrogel-based interface regulation, the skin compatibility of conductive materials is also important. Ahn et al. [92] achieved a balance between skin compatibility and electrical conductivity using PEDOT:PSS electrodes, with no detectable skin irritation during α/β-wave experiments.

As neural interfaces extend from epidermal contact toward cortical and intracerebral tissues, the focus of biocompatibility shifts from skin tolerance to regulation of the immune microenvironment and tissue integration. Owing to their favorable tissue affinity and tunable structural properties, natural biomaterials are increasingly being explored to construct more compliant neural interfaces. For example, Hu et al. [93] stably integrated a predefined conductive layer into a silk fibroin (SF) substrate to construct a multichannel flexible SF neural interface and demonstrated its feasibility for ECoG recording together with a tendency toward reduced inflammatory responses in a rat model (Fig. 3A). The hydrated compliance and tissue affinity of SF facilitated conformal contact with the cortical surface (Fig. 3A, I), while structural integration with the conductive layer improved interfacial integrity and reduced micromotion-induced disturbance (Fig. 3A, II and III). Correspondingly, blood interferon-γ (IFN-γ) levels in the SF-based interface groups were lower than those in the PI patch group at 7, 14, and 28 d post-implantation and closer to those in the sham group (Fig. 3A, IV), supporting its low immune activation profile. However, these brain-surface interfaces have primarily been evaluated for relatively short-term local inflammatory responses, while their ability to regulate long-term mechanical stimulation and chronic glial responses under deep implantation remains to be further validated. To address this limitation, Wang et al. [94] developed an 8-μm-thick serpentine neural probe using a flexible Parylene C substrate, with oxygen-plasma treatment and an SF–poly(ethylene glycol) (PEG) coating to improve tissue compatibility. The low stiffness of Parylene C reduced mechanical mismatch with brain tissue, while surface hydrophilization decreased the contact angle from approximately 90° to 7.8°. After 9 months of implantation, microglial and astrocytic accumulation around the probe remained markedly lower than that around silicon-based electrodes, indicating that material compliance combined with surface engineering reduced chronic inflammatory responses. Overall, biocompatibility in BCI interfaces has expanded from material cytocompatibility to multidimensional design involving antibacterial properties, breathability, mechanical matching, and immune modulation. Nevertheless, most studies still focus on individual interfacial factors or limited implantation periods. Maintaining material stability, tissue homeostasis, and signal reliability simultaneously in long-term dynamic environments remains a key challenge for chronic BCI applications.

**Fig. 3.**
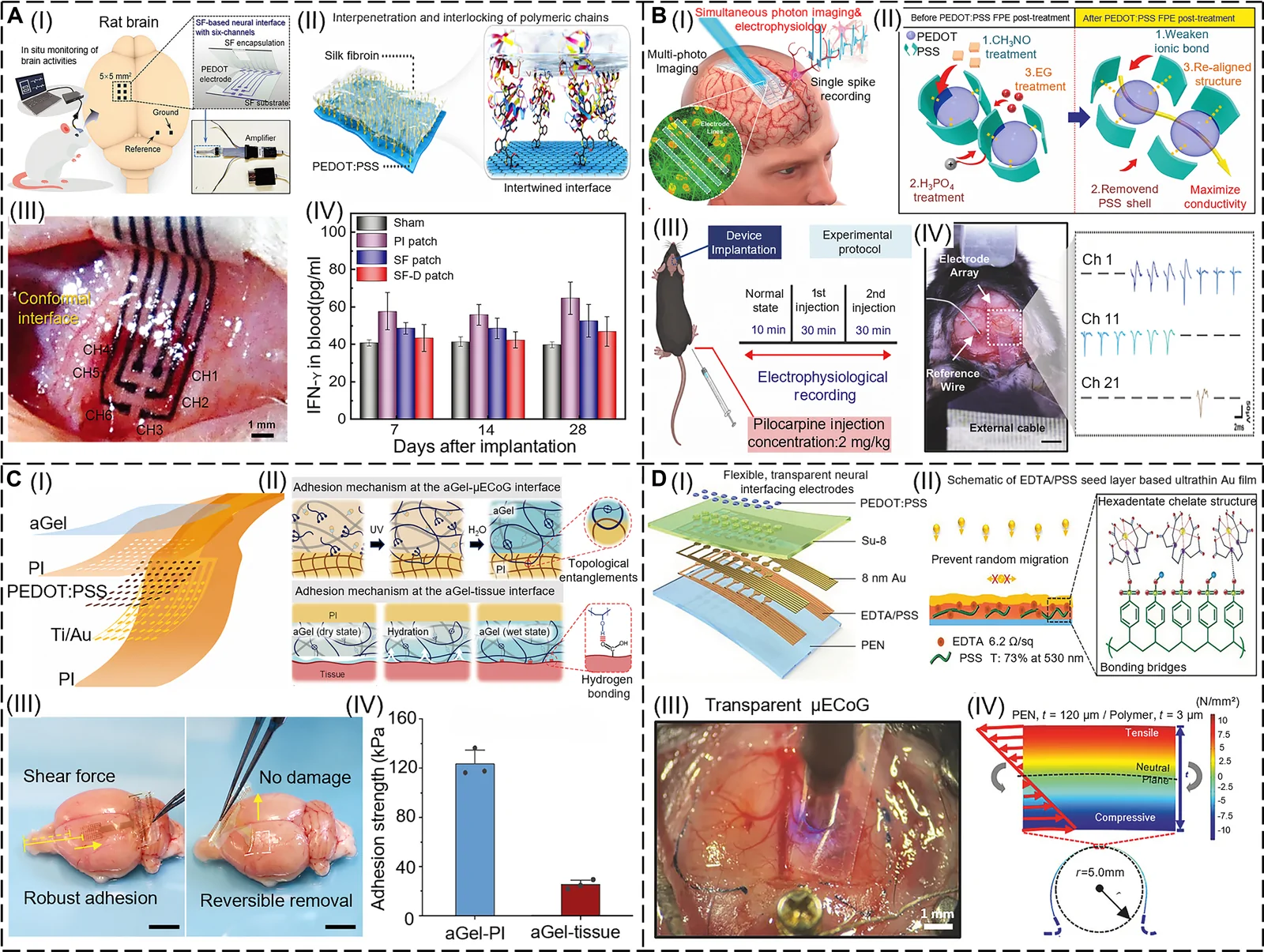
Key performance requirements of neural interfaces for BCIs supporting information reliability. (A) Biocompatibility of neural interfaces for BCIs: (I) Schematic illustration of SF-based biocompatible BCI interface design; (II) material architecture of biocompatible BCI electrodes; (III) photograph of the SF-based neural electrode conformally interfaced with the cortical surface; (IV) blood interferon-γ (IFN-γ) levels measured at 7, 14, and 28 d post-implantation. Reproduced from Ref. [93] with permission from Wiley-VCH GmbH. Copyright © 2024 Wiley-VCH GmbH. (B) Electrical and electrochemical performance of neural interfaces for BCIs: (I) Schematic overview of electrically and electrochemically functional BCI interfaces; (II) conductive mechanism of PEDOT within BCI electrodes; (III) experimental schematic of PEDOT conductive BCI in a pilocarpine-induced epilepsy mouse model; (IV) implantation of PEDOT conductive BCI on the mouse cortex and electrophysiological signal recording. Reproduced from Ref. [103] under the CC BY-NC-ND 4.0 license. Copyright © 2025 The Author(s). (C) Interfacial adhesion of neural interfaces for BCIs: (I) structural design of the bioadhesive aGel-μECoG interface; (II) interfacial adhesion mechanism of aGel-μECoG; (III) photographs demonstrating robust tissue adhesion and reversible removal of aGel-μECoG; (IV) evaluation of the interfacial adhesive performance of aGel-μECoG. Reproduced from Ref. [113] under the CC BY 4.0 license. Copyright © 2025 The Author(s). (D) Mechanical matching and fatigue reliability of neural interfaces for BCIs: (I) structural design of multilayer ultrathin flexible μECoG; (II) mechanical stabilization mechanism of multilayer ultrathin flexible μECoG; (III) mechanical conformability of multilayer ultrathin flexible μECoG on tissue surfaces; (IV) simulated stress distribution of the multilayer ultrathin flexible μECoG structure. Reproduced from Ref. [118] under the CC BY 4.0 license. Copyright © 2025 The Author(s).

### Electrical and electrochemical performance

Electrical and electrochemical properties determine the efficiency of ion-to-electron signal transduction in BCI neural interfaces and are key determinants of signal fidelity and stimulation safety within the information reliability chain [95,96]. BCI electrodes require not only high electronic conductivity but also low interfacial impedance, low noise, high charge storage/injection capability, and stable long-term polarization behavior [97]. In neural recording, low impedance and high effective surface area contribute to improving the SNR of weak neural signals. In electrical stimulation and bidirectional closed-loop BCIs, stable charge transfer capability is closely related to stimulation efficiency, tissue safety, and long-term electrochemical durability [98]. Therefore, conductive performance should not be evaluated solely by the intrinsic conductivity of materials but should be comprehensively assessed based on electrode–tissue interfacial impedance spectra, noise level, charge transfer capability, and long-term stability. Carbon-based materials have been widely applied to reduce interfacial impedance in neural electrodes because of their continuous π-conjugated structures, high specific surface area, and favorable processability for flexible devices. Dong et al. [99] developed flexible neural electrodes based on reduced graphene oxide (rGO), in which hydroiodic acid reduction removed oxygen-containing functional groups and restored the sp^2^-conjugated network to enhance electron transport. The porous rGO-3D network further promoted neural tissue ingrowth and close electrode–tissue integration. rGO-3D exhibited an interfacial impedance of 2,024 ± 134 Ω·cm^2^ at 1 kHz and enabled continuous neural recording in freely moving rats. Spontaneous LFPs across different frequency bands were stably recorded at 7, 12, 14, and 21 d after implantation, with no significant differences from silver-wire controls. However, single electronic conduction networks still involve a trade-off between electrical performance and long-term tissue integration, making coupled ion–electron transport a potential strategy for improving chronic recording performance. Wang et al. [100] combined rGO with PEDOT:PSS to construct an ultraflexible electrode with coupled electronic–ionic transport. rGO provided efficient electronic pathways, while PEDOT:PSS enhanced interfacial ion–electron coupling, enabling both low impedance and broadband signal transmission. The electrode simultaneously supported dopamine detection and broadband neural recording in deep-brain regions and maintained stable electrochemical and electrophysiological monitoring for 6 weeks in vivo, indicating that composite conductive networks can improve long-term recording stability.

As recording resolution advances toward the single-neuron level, weak neural signals become increasingly sensitive to interfacial impedance and noise, placing greater demands on charge-transfer efficiency. Kondrataviciute et al. [101] uniformly deposited Ti_3_C_2_T_X_ MXene onto carbon-fiber electrodes, using its high conductivity and large specific surface area to enhance interfacial charge transfer. This reduced electrode resistance from 3 to 6 kΩ to 78 to 147 Ω, with a conductivity of 1.25 × 10^5^ S·m^−1^. After implantation in the rat hippocampal CA1 region, the interfacial impedance remained stable at approximately 250 kΩ over 4 weeks, whereas that of uncoated carbon electrodes increased from approximately 1 to 2 MΩ. The 60-Hz line noise also remained stable, and single-neuron recording was achieved. As BCIs evolve from unidirectional recording toward integrated recording, stimulation, and feedback modulation, electrode materials must also combine low impedance, high charge-injection capability, and long-term electrochemical stability. Wang et al. [102] developed an ultraflexible bidirectional neural probe modified with PEDOT:PSS/IrOx, in which PEDOT:PSS provided a highly conductive, low-impedance interface for neural signal transmission, while IrOx enhanced electrochemical charge-storage capacity. The composite modification reduced the impedance at 1 kHz from 3.47 ± 1.77 MΩ to 41.88 ± 4.04 kΩ and increased the charge-storage capacity from 0.14 ± 0.01 mC cm^−2^ to 24.75 ± 0.18 mC cm^−2^, expanding the applicability of bidirectional neural interfaces to behavioral modulation and closed-loop BCIs. However, this composite interface was primarily designed for electrophysiological recording and stimulation, while its opacity limits integration with optical imaging. To address this limitation, Kim et al. [103] developed a transparent, metal-free neural interface based on highly conductive PEDOT. A 3-step formamide, phosphoric acid, and ethylene glycol (FPE) treatment (CH₃NO–H₃PO₄–EG) weakened PEDOT–PSS ionic interactions, removed insulating PSS, and shortened the π–π stacking distance of PEDOT, thereby reconstructing conductive pathways. The resulting conductivity reached 3.4 × 10^3^ S·cm^−1^, while 20 × 20 μm^2^ microelectrodes exhibited an impedance of only 45.8 kΩ at 1 kHz. In vivo, the root-mean-square (RMS) noise was 6.6 to 6.8 μV, markedly lower than the 50 to 60 μV of indium tin oxide electrodes, while single-neuron firing under normal and seizure-induced conditions could be stably distinguished (Fig. 3B, I to IV), demonstrating that PEDOT:PSS microstructural regulation can improve weak neural signal recording while maintaining optical transparency. Conductive interfaces are further evolving from static electrical optimization toward stimulus-responsive dynamic regulation. Dutta et al. [50] balanced thermoreversibility and continuous charge transport by regulating the phase morphology of PEDOT:PSS/ionic biogels. An interconnected PEDOT network provided continuous electronic transport pathways, enabling the bicontinuous marginally percolated (BC-MP) biogel to achieve a conductivity of approximately 7.7 S·cm^−1^ at 60 °C while maintaining a low storage modulus of approximately 1.2 kPa. The interface maintained a low contact impedance of approximately 1.6 kΩ·cm^2^ for more than 3 d under different hair conditions. After 10 d of repeated use, the impedance of BC-MP increased by only approximately 500 Ω·cm^2^, compared with approximately 1,500 Ω·cm^2^ for the ion-conduction-dominated nucleated system, indicating that phase-morphology regulation can balance thermoresponsive interfacial adaptability and long-term electrical stability. Overall, conductive design of BCI neural interfaces is progressing from single electronic conduction toward coupled ion–electron transport and dynamic responsive regulation. Nevertheless, simultaneously maintaining low impedance, efficient charge transfer, and long-term electrochemical stability in complex in vivo environments remains a key challenge for long-term high-fidelity recording and safe stimulation.

### Interfacial adhesion

In BCI neural interfaces, adhesion is not merely required to prevent electrode detachment but is a key determinant of maintaining stable contact with the skin, meninges, or brain tissue [104,105]. Low interfacial impedance and efficient charge transfer can only be translated into high-fidelity neural signals under stable interface conditions. Relative sliding, local delamination, or fluid infiltration at the electrode–tissue interface may induce rapid impedance fluctuations and further amplify motion artifacts and baseline drift [106]. Among available interface materials, conductive hydrogels have been widely adopted in flexible EEG electrodes because of their high-water content, tissue-like mechanical properties, and efficient ionic transport capability. These characteristics enable the formation of soft conformal interfaces without relying on strong external adhesives [107]. To improve adhesion stability under wet and dynamic conditions, strategies based on covalent crosslinking, dynamic bonding, and multiple weak interactions are commonly introduced to strengthen interfacial integration. Yang et al. [108] developed an injectable conductive adhesive hydrogel based on a PEG network, in which tannic acid synergistically regulated thiol–maleimide click chemistry and multiple noncovalent interactions to enhance wet-tissue adhesion through interfacial dehydration, hydrogen bonding, and covalent anchoring. The hydrogel achieved an adhesion strength of 20.9 kPa to neural tissue, with an interfacial toughness approximately 5.3-fold higher than that of the low-solid-content system. However, strong tissue adhesion alone cannot prevent interfacial failure between the conductive layer and device substrate, and delamination under long-term deformation may also disrupt electrical continuity. To address this limitation, Tian et al. [109] constructed an interpenetrating network using a polydopamine (PDA) adhesive layer and PEDOT-MeOH:PSS conductive polymer, where PDA-mediated interfacial anchoring and internetwork hydrogen bonding strengthened the adhesion between the conductive layer and metal electrode. After 20 min of ultrasonication at 500 W, 93% of the coating area was retained. These findings indicate that adhesive design should consider the stability of both the electrode–tissue and conductive layer–device interfaces, rather than focusing solely on high tissue adhesion strength.

Motor rehabilitation scenarios further amplify the risk of adhesion failure. Hair barriers, sweat electrolytes, sebum secretion, repeated deformation, and prolonged wear can weaken effective electrode–skin contact, resulting in intermittent signal loss and increased artifacts. Therefore, adhesive interfaces should provide not only sufficient initial bonding strength but also environmental adaptability and dynamic compliance. Hsieh et al. [110] developed a self-adhesive AIRTrode hydrogel electrode based on poly(2-acrylamido-2-methylpropanesulfonic acid) (PAMPS), PEDOT:PSS, and glycerol. Hydroxyl, sulfonic acid, and amide groups formed multiple hydrogen bonds with the stratum corneum, while the flexible interface increased the effective contact area for adaptive adhesion. The electrode achieved a skin adhesion force of 0.92 ± 0.21 N cm^−1^ and remained stable after 20 adhesion–peeling cycles, improving skin–electrode interface stability under hair-covered and dynamic conditions. Although reversible hydrogen bonding facilitates repeated attachment and dynamic adaptation, noncovalent interactions alone may still permit interfacial slippage under greater friction, sweating, and large deformation. Kim et al. [111] developed a PVA/reduced graphene oxide (RGO)/poly(acrylic acid) (PAA)–N-hydroxysuccinimide (NHS) hydrogel electrode, in which electrostatic interactions of PAA carboxyl groups and NHS ester-mediated covalent bonding enhanced skin adhesion, while RGO maintained conductive pathways. The electrode achieved an adhesion strength of 6.5 kPa on the forearm and remained stable after 10 adhesion–peeling cycles, enabling stable EEG acquisition during movement and sweating. However, stable skin–electrode adhesion cannot fully prevent connection instability caused by mechanical mismatch between flexible electrodes and external acquisition devices. Adhesive design should therefore extend from the skin interface to stable system-level interconnection. To this end, Zhang et al. [112] developed a dry electrode that could be directly coated onto the skin, using ultraconformal contact and a thickness gradient to reduce interfacial stress. The electrode achieved a contact impedance of 10.8 kΩ·cm^2^ and a skin adhesion strength of approximately 963 kPa. A porous silver-fabric connector provided mechanical interlocking and a modulus gradient, improving connection stability between the flexible electrode and external devices. The platform further enabled through-hair EEG neural response detection without obvious magnetic resonance imaging (MRI) artifacts, demonstrating potential for stable neural monitoring and multimodal MRI–electrophysiological integration.

As the recording system extends to implantable ECoG, adhesive design shifts from preventing detachment toward stable, low-damage fixation under brain pulsation and in the presence of tissue fluids. Chen et al. [113] developed a PVA/poly(3-(trimethoxysilyl)propyl methacrylate) (PTPM) heterogeneous-network aGel and integrated it with a μECoG array (Fig. 3C). On the electrode side, stable physical fixation was achieved through topological interlocking with the PI substrate, whereas on the tissue side, the hydrated soft network formed multiple weak interactions with the cortical surface (Fig. 3C, I and II). This dual-interface adhesion mechanism enhanced fixation while avoiding excessive mechanical constraint associated with simply increasing adhesive strength. Peeling, reversible removal, and adhesion tests further demonstrated that the interface maintained strong adhesion while supporting low-damage detachment and exhibited a reduced tendency toward fibrous capsule formation, supporting its potential for chronic application from both interfacial stability and tissue response perspectives (Fig. 3C, III and IV). Overall, adhesive design for BCI interfaces has progressively shifted from pursuing high adhesion strength alone toward the coordinated regulation of wet-state stability, dynamic adaptability, and low-damage fixation. However, balancing stable adhesion, interfacial compliance, and tissue safety in complex physiological environments over the long term remains a key challenge for reliable chronic neural interfaces.

### Mechanical matching and fatigue reliability

Following stable interfacial contact, BCI electrodes must maintain low tissue disturbance and structural integrity under long-term micromotion, bending, twisting, and cyclic loading. The mechanical performance of BCI electrodes is not determined solely by elastic modulus but by effective compliance arising from the combined effects of modulus, thickness, multilayer architecture, and geometric design, particularly bending stiffness and bending compliance [22]. When electrode stiffness substantially exceeds that of brain tissue, interfacial loads become difficult to dissipate uniformly, and localized stress concentration together with micromotion-induced shear may trigger tissue responses, impedance drift, and signal attenuation [114]. Therefore, the objective of mechanical matching is not simply to reduce modulus but to regulate stress transfer, preserve conductive continuity, and delay fatigue failure during long-term operation. For cortical ECoG interfaces, electrodes must first accommodate brain pulsation and local surface deformation. Ji et al. [115] developed 3-dimensional flexible micro-bump electrodes with a height of approximately 327 μm on a PI substrate. PI provided flexible support, while soft silicone micro-bumps and micro-slits accommodated brain-surface deformation through elastic buffering and stress release, reducing interfacial stress by 59.2% at 50% compressive strain. PEDOT:PSS modification further reduced interfacial impedance. After 1,000 compression cycles, the impedance at 1 kHz remained stable at approximately 1.80 kΩ, although the charge-storage capacity decreased by 51.2%. These results indicate that coordinated material and structural design can maintain interfacial conformity and electrical continuity under brain pulsation and local deformation, supporting stable ECoG recording. However, such local 3-dimensional buffering structures primarily address normal compression and local stress concentration, with limited adaptability to overall device bending and deformation along complex geometries. To improve overall structural flexibility, Hong et al. [116] developed a monolayer PEDOT:PSS open-mesh brain electrode on a 25-μm-thick flexible polyethylene terephthalate (PET) substrate. The flexible substrate and micropatterned mesh enhanced bending adaptability, while PEDOT:PSS provided a low-impedance conductive interface. Although the open-mesh area was only approximately 30% of that of the planar electrode, its impedance at 1 kHz remained 19.34 kΩ, close to 14.64 kΩ for the planar electrode, indicating that mesh-based material reduction can improve structural flexibility while maintaining the electrical performance required for neural recording. For long-path in vivo deployment, Wang et al. [117] developed an ultraflexible uFINE-I neural electrode using a 5-μm-thick PI substrate. The high tensile strength of PI combined with the ultrathin structure provided both flexibility and structural integrity, enabling navigation and implantation along tortuous vascular pathways of 330 to 350 mm. After implantation, the electrode stably recorded LFPs and single-neuron spikes, with a mean spike amplitude of 76.08 μV and an SNR of 5.42.

However, as neural interfaces become increasingly ultrathin and transparent, metal-layer cracking, interfacial stress concentration, and degradation of electrical continuity under repeated deformation have emerged as key limitations to long-term mechanical reliability. To address this issue, Kim et al. [118] developed a transparent ultrathin μECoG based on an EDTA/PSS hexadentate metal-complex seed layer, comprising a polyethylene naphthalate (PEN) substrate, EDTA/PSS seed layer, 8-nm gold (Au) conductor, SU-8 encapsulation layer, and PEDOT:PSS contact layer. Stable coordination between EDTA and Au, together with PSS-enhanced interfacial bonding and continuous Au-film formation, reduced stress concentration and crack propagation (Fig. 3D, I to IV). The ultrathin Au exhibited only an approximately 15% increase in resistance at ~150% strain and only minor microcracking after 50% stretching. After 50,000 bending cycles at a 5-mm radius, the resistance increase remained below 0.05%, with no obvious cracking. Furthermore, during a 2-week in vivo study, the increase in RMS noise on day 14 relative to day 7 remained below 10%, indicating that interfacial coordination combined with an ultrathin conductor can maintain structural integrity, electrical continuity, and ECoG recording stability under repeated mechanical deformation. However, this strategy primarily improves the crack resistance and cyclic deformation stability of ultrathin metal conductors, and its interfacial and electrical stability under longer-term in vivo conditions and multiaxial mechanical loading requires further validation.

Overall, the long-term reliability of BCI neural interfaces is not determined by a single material property but depends on the coordinated regulation of biocompatibility, electrical and electrochemical transport, interfacial adhesion, and mechanical matching. By reducing tissue responses and interfacial impedance, maintaining stable contact, and mitigating long-term micromotion and fatigue failure, material and structural design can reduce signal attenuation, increased noise, and channel instability, thereby maintaining continuous and stable input at the upstream end of the information reliability chain. Accordingly, neural interface materials should progress from single-property optimization toward multiproperty coordination and long-term stability, providing high-quality input for reliable AI-based decoding of nonstationary neural signals.

## AI-Driven Neural Decoding and Reliable Information Transfer

Stable neural signals enabled by advances in neural interface materials and structural engineering provide a reliable input foundation for BCI decoding. However, long-term rehabilitation is inevitably accompanied by fluctuations in neural activity, intersession and intersubject variability, limited training data, and changes in channel conditions, through which upstream signal perturbations can propagate into feature drift and decoding uncertainty [119]. Within the information reliability chain, therefore, the role of AI extends beyond improving instantaneous classification or prediction accuracy. More importantly, AI can mitigate the propagation of upstream signal variability into decoding outputs through data-efficient learning, spatiotemporal and relational representation of neural activity, adaptation to distribution shifts, and computationally efficient real-time decoding. Accordingly, this section focuses on key reliability challenges associated with limited neural data, complex neural information representation, neural distribution shifts, and real-time closed-loop operation while comparing how different AI approaches address these challenges and support the stable transfer of neural information from signal input to motor-intent output (Table S1).

### Data-efficient learning with limited neural data

BCI model training for motor assistance and neurorehabilitation is often constrained by limited data availability. For patients with stroke, SCI, or severe motor impairment, restricted effective training time further complicates the acquisition of sufficiently labeled data [120]. Under these conditions, AI approaches can improve neural data utilization through 3 complementary strategies: limited-sample discrimination, label-efficient representation learning, and data augmentation, thereby reducing the impact of data scarcity on motor-intent decoding reliability. With limited labeled data, support vector machines (SVMs) maximize class margins to establish discriminative boundaries and, combined with engineered representations such as common spatial patterns (CSPs) and time–frequency features, enable movement-related EEG classification at relatively low training cost [121,122]. SVMs thus provide a lightweight decoding pathway by establishing a relatively stable feature-to-intent decision boundary (Fig. 4A). In a 4-class motor-imagery EEG dataset from 62 healthy participants, Belwafi and Ghaffari [123] applied common average reference (CAR)/Laplacian filtering and extracted power spectral density (PSD), Hjorth parameters, autoregressive coefficients, entropy, and higher-order statistical features. A linear SVM with a one-versus-rest strategy achieved 78.82% accuracy, 78.0% precision, and a 77.0% F1 score. However, stable initial discrimination does not ensure sustained adaptation to changes in neural signal distributions. Dynamically adjusting the decision boundary as new data become available is therefore important for addressing intersession variability. Thanigaivelu et al. [124] employed an incremental SVM (ISVM) that updates the decision boundary with newly acquired EEG samples without complete retraining, achieving a mean classification accuracy of 92.24% on BCI Competition III dataset 4a and session-wise accuracies of 92.45% and 94.57% on BCI Competition IV datasets 2a and 2b, respectively. Although incremental learning enhances adaptation to newly acquired and intersession data, dependence on handcrafted features still limits SVMs in modeling complex spatiotemporal neural representations and continuous motor states.

**Fig. 4.**
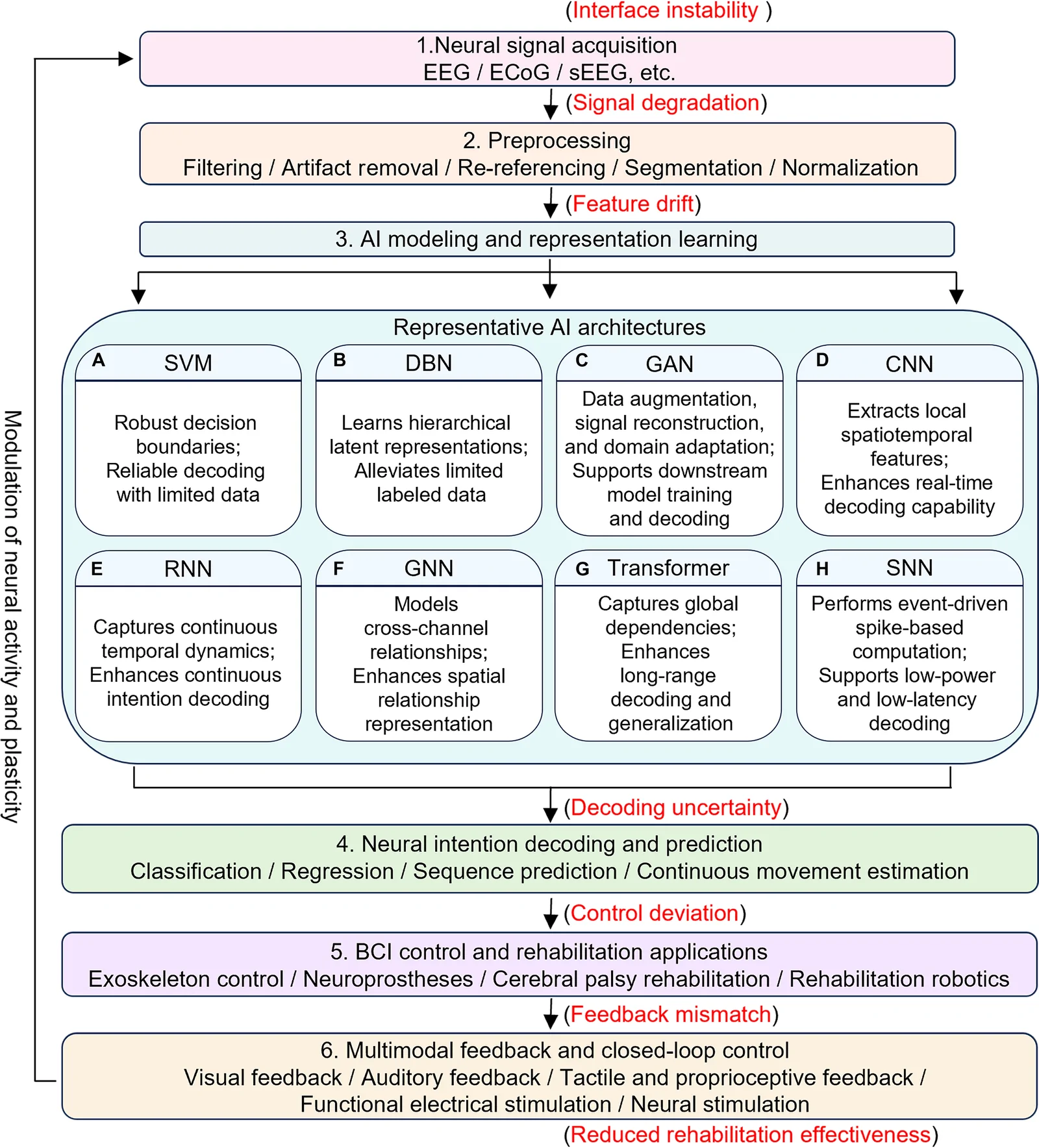
Neural information processing workflow, representative AI architectures, and failure propagation pathway in AI-enabled BCIs. Neural information sequentially undergoes neural signal acquisition, preprocessing, AI modeling and representation learning, neural intention decoding and prediction, BCI control and rehabilitation applications, and multimodal feedback and closed-loop control. Representative AI architectures include (A) SVM, (B) DBN, (C) GAN, (D) CNN, (E) RNN, (F) GNN, (G) Transformer, and (H) SNN.

When unlabeled neural data are relatively abundant, data-efficient learning can extend from discriminative boundary optimization to latent representation learning with reduced label dependence. Deep belief networks (DBNs) learn latent features layer by layer through stacked restricted Boltzmann machines and construct hierarchical representations via “unsupervised pretraining–supervised fine-tuning” (Fig. 4B) [125,126], thereby reducing dependence on large-scale labeled data. In an EEG-based epilepsy detection task, Qiao et al. [127] combined a convolutional DBN with self-attention and dual-task learning, using stacked CssCRBMs to learn hierarchical representations and capture long-range temporal dependencies, achieving a detection accuracy of 94.1%. However, the reported performance was obtained using a hybrid architecture and cannot be attributed to the DBN component alone. Wang et al. [128] further optimized a DBN using the sparrow search algorithm (SSA) and combined it with empirical mode decomposition (EMD) to extract movement-related EEG features. The resulting SSA-DBN achieved classification accuracies of 86.14% to 87.83% across multiple datasets, improving upon the standard DBN by 5.57% to 10.38%.

Whereas SVMs and DBNs primarily improve the utilization of existing limited data, generative adversarial networks (GANs) further augment or reconstruct training data at the data level. Through adversarial learning between a generator and a discriminator, GANs approximate real neural data distributions and can support sample generation, signal reconstruction, and domain adaptation for downstream BCI decoding (Fig. 4C) [129,130]. Data augmentation represents the most direct application for alleviating limited-sample constraints. Song et al. [131] proposed EEGGAN-Net, using a conditional GAN (CGAN) to generate motor-imagery EEG samples for joint classifier training with real data. Maximum classification accuracies reached 81.3% and 90.3% on BCI Competition IV-2a and IV-2b, respectively; CGAN ablation reduced both accuracy and stability, supporting the benefit of generative augmentation for limited-sample decoding. Beyond sample quantity, generated-data quality also determines downstream value. Du et al. [132] proposed L-C-WGAN-GP, using a long short-term memory (LSTM) generator to learn temporal dependencies in EEG and combining a convolutional neural network (CNN) discriminator with a gradient-penalized Wasserstein distance to improve adversarial training stability. Augmentation with generated data reduced EEG compressed-sensing reconstruction loss by approximately 15% compared with using only the original data. However, generated samples may introduce noise or spurious features, and statistical similarity to real EEG does not necessarily imply neurophysiological consistency. Generative bias may therefore propagate along the information reliability chain to downstream decoding. Overall, SVMs, DBNs, and GANs address BCI neural data scarcity through complementary strategies of limited-sample discrimination, label-efficient representation learning, and data augmentation, respectively. However, they remain constrained by dependence on handcrafted features, limited capacity for complex spatiotemporal representation, and uncertain neurophysiological consistency of generated data, indicating that improved data utilization efficiency does not necessarily translate into stable decoding reliability.

### Spatiotemporal and relational representation of neural signals

Motor-intent information is distributed across multiple neural channels and temporal scales, while noise, interindividual variability, and changes in neural states can perturb these spatiotemporal patterns, making it difficult for decoders relying on fixed handcrafted features to maintain stable representations [133]. To address this challenge, CNNs, recurrent neural networks (RNNs), and graph neural networks (GNNs) extract movement-related neural information from 3 complementary perspectives: local spatiotemporal patterns, continuous temporal dynamics, and interchannel spatial relationships, respectively. CNNs can directly learn local spatiotemporal features from multichannel neural signals, including EEG and ECoG, reducing dependence on predefined feature engineering and providing discriminative representations for motor-intent decoding (Fig. 4D) [134,135]. Borra et al. [136] used a lightweight interpretable CNN (MS-Sinc-ShallowNet) to continuously decode 2-dimensional hand position and velocity from EEG recordings of 13 participants, identifying movement-related features predominantly in the delta band and central–parietal regions. With only 2 to 10 training trials, cross-subject transfer increased the number of participants achieving significant decoding from 4 to 9; however, this advantage was no longer significant with 20 to 50 trials, indicating that the benefit was primarily confined to low-data conditions and was influenced by individual data availability and signal representation quality. Extending continuous movement representation to real-time fine motor control further requires robustness to temporal signal variations and stable continuous prediction. Ding et al. [8] applied the CNN-based EEGNet to real-time closed-loop decoding of fine motor intentions. In 21 participants, online training and fine-tuning yielded real-time decoding accuracies of 80.56% and 60.61% for 2- and 3-finger motor imagery, respectively, while same-day fine-tuning and online smoothing reduced label switching during continuous prediction. However, 3-class performance remained substantially lower than binary classification and increasing network capacity improved performance by only ~1.5%, suggesting that fine motor decoding remains constrained by low SNR, limited spatial resolution, and overlapping neural representations.

Although CNNs capture local spatiotemporal patterns, continuous motor intentions also involve neural states that evolve over time, and local receptive fields alone may not fully characterize such sequential dependencies. RNNs integrate historical neural activity through hidden-state propagation across successive time steps, extending motor-intent decoding to continuous temporal dynamics (Fig. 4E) [137,138]. Deo et al. [139] applied an RNN to continuous bimanual movement decoding in individuals with paralysis, mapping cortical neural activity to real-time dual-cursor control. In offline single-hand decoding, mean correlation coefficients reached 0.90 and 0.83 for the right and left hands, respectively. However, direct online deployment produced pulsatile outputs; temporal scaling and reordering of training sequences yielded more continuous control, highlighting a remaining gap between effective temporal modeling and stable online control. As neural sequences become longer, conventional RNNs progressively lose the ability to retain earlier states. LSTM networks selectively retain and update historical information through gating mechanisms, reducing the loss of long-range temporal information during recurrent propagation [140,141]. Martín-Chinea et al. [142] applied an LSTM to real-time BCI classification using low-cost wireless EEG. As the temporal window increased from 1 to 7 s, mean classification accuracy increased from 76.61% to 90.94%, whereas further extension produced no substantial improvement. These findings reveal a boundary in temporal information utilization: Accumulating historical information can strengthen temporal representations, but continuous BCI operation requires a trade-off between information utilization and response latency.

Beyond temporal dynamics, movement-related neural activity involves spatial coordination and functional connectivity across electrodes and brain regions, which cannot be fully captured by local convolution or sequential propagation. GNNs represent neural channels or brain regions as nodes and aggregate interchannel information through their connections, enabling explicit modeling of non-Euclidean spatial relationships (Fig. 4F) [143,144]. Tang et al. [145] proposed spatial filter temporal graph convolutional network (SF-TGCN), which uses Laplacian spatial filtering to reduce inter-electrode redundancy and graph convolution to learn interchannel topological relationships in motor-imagery EEG. Mean classification accuracies reached 80.82% and 87.98% on BCI Competition IV-2a and an in-house dataset, respectively. Replacing graph convolution with conventional CNN-based channel convolution reduced accuracy by 1.17 and 1.82 percentage points, respectively, directly demonstrating the contribution of interchannel topology modeling to movement-related spatial representations. Moreover, different interchannel connections contribute unequally to motor-intent decoding. Leng et al. [146] constructed interchannel functional connectivity using phase-locking value (PLV) and extracted time–frequency features using continuous wavelet transform (CWT) in 10 patients with SCI, while graph attention networks (GATs) and gated recurrent units (GRUs) were used to model connection weights and temporal dynamics, respectively. TFSGAT achieved a mean classification accuracy of 95.82 ± 0.86%, exceeding those of GCN (81.56 ± 2.13%) and GAT (85.58 ± 1.74%); however, accuracy decreased to 90.55% under leave-one-subject-out validation. This decline exposes a reliability boundary of graph-based relational representations: Even when the model adaptively differentiates the importance of interchannel connections, interindividual EEG variability can still weaken cross-subject generalization. Overall, CNNs, RNNs, and GNNs enhance the representation of movement-related neural information through complementary mechanisms of local spatiotemporal feature extraction, continuous temporal dynamics modeling, and interchannel relational representation, respectively. However, their reliability remains jointly constrained by signal quality, temporal information utilization, and variations in spatial relationships. Richer neural representations must therefore ultimately translate into stable decoding performance across individuals, sessions, and real-time operating conditions.

### Generalization and adaptation under neural distribution shifts

Neural signals are inherently nonstationary, and variations across subjects, recording sessions, and neural states can alter the statistical distributions of movement-related features, leading to performance degradation when existing models are applied to new subjects or sessions [147]. Through self-attention, Transformers capture global dependencies across time steps and neural features and can further integrate cross-domain information exchange and feature alignment, providing a new modeling pathway for generalization and adaptation under neural distribution shifts (Fig. 4G) [148,149]. Addressing such shifts first requires reducing dependence on fixed local features and extracting relatively st able discriminative information over longer temporal ranges. Luo et al. [150] proposed a shallow mirror Transformer (SMT) that uses multi-head self-attention to identify and integrate critical temporal segments across entire EEG trials. In cross-subject validation on BCI Competition IV-2a, IV-2b, and OpenBMI, the model achieved mean accuracies of 74.48% and 76.10% for new and existing subjects, respectively. The remaining performance gap indicates that global information integration can mitigate variations in discriminative temporal segments but cannot eliminate distribution shifts arising from interindividual differences. Whether such representations remain stable over time must therefore be further evaluated. Zhang et al. [151] proposed a local–global convolutional Transformer integrating local and global Transformers with CNNs to capture spatiotemporal information. Under 2 cross-session settings on the Korea University dataset, the model improved performance over comparison methods by up to 7.49% and 8.19%, respectively, and by 1.24% and 2.12% on BCI Competition IV-2a. These results extend the evaluation of Transformer reliability from a single data distribution to cross-session scenarios; however, the reported gains were obtained from predefined offline sessions and therefore do not represent continuous adaptation during long-term use.

Beyond cross-session stability, performance in completely unseen subjects further reflects cross-subject generalization. Keutayeva et al. [152] proposed a compact convolutional Transformer (EEGCCT), using convolution to extract local spatiotemporal features and a Transformer encoder to model global correlations. In leave-one-subject-out (LOSO) evaluation on BCI Competition IV-2b, EEGCCT-2 achieved a mean accuracy of 70.12%, compared with 68.42% for EEGNet and 69.72% for Conformer. However, the improvement remained limited, suggesting that enhanced global representation alone is insufficient to fully overcome interindividual differences in EEG distributions. When representation learning and model generalization alone cannot compensate for such distribution differences, a more direct strategy is to actively reduce feature shifts between the source and target domains. Zhang et al. [153] proposed the Transformer-based domain adaptation network MI-CAT, treating data from other subjects and the current subject as the source and target domains, respectively. Self-attention and cross-attention were used for intra- and interdomain information exchange, while cross-Transformer blocks (CTBs) enabled feature alignment. The model achieved mean classification accuracies of 85.26% and 76.81% on BCI Competition IV datasets IIb and IIa, respectively. Ablation analyses further showed that incorporating source-domain information and cross-attention improved performance, while using nonshared attention blocks for the source and target domains yielded better overall performance. However, increasing cross-domain interaction did not continuously improve performance; greater CTB depth resulted in performance degradation, suggesting that excessive interdomain information transfer may introduce redundant features and increase the risks of negative transfer and overfitting. Overall, Transformers have extended BCI decoding from global feature modeling to cross-session generalization and cross-domain adaptation. However, improved offline performance does not necessarily translate into long-term reliability. Achieving continuous adaptation to neural distribution shifts while reducing repeated calibration therefore remains a key challenge for long-term BCI applications.

### Efficient decoding for real-time closed-loop operation

For real-time closed-loop BCIs, stable neural representations and accurate decoding must ultimately be translated into continuous control outputs with low latency and computational overhead. Particularly in wearable or portable systems, neural signals must be continuously processed under constrained computational and energy resources, making computational efficiency, energy consumption, and response latency key constraints in the transition from offline decoding to real-time closed-loop operation [154]. Spiking neural networks (SNNs) transmit information through discrete spikes and their timing and employ event-driven computation, thereby reducing unnecessary continuous numerical operations for sparse inputs. This provides a potentially efficient computational paradigm for resource-constrained BCIs and deployment on neuromorphic hardware (Fig. 4H) [155,156]. Reduced computational and energy costs are important advantages of SNNs for real-time operation. Zhang et al. [157] developed a lightweight SNN that extracts motor imagery electroencephalogram (MI-EEG) features through spatiotemporal convolution and uses trainable leaky integrate-and-fire (LIF) neurons to encode continuous neural activity into sparse spike sequences. Across 3 public MI datasets, the model achieved a mean within-subject classification accuracy of 69.19%, with an estimated mean energy consumption of only 24 μJ, at least ~50% lower than those of EEGNet, FBCNet, ShallowNet, and DeepNet, demonstrating the potential of SNNs for lightweight decoding in resource-constrained BCIs.

While reducing computational overhead, whether spike encoding preserves effective neural information further determines the balance between computational efficiency and decoding reliability. Lutes et al. [158] combined convolutional feature extraction with spike-based computation to construct a convolutional spiking neural network (CSNN) for intention detection from movement-related anticipatory slow cortical potentials in EEG. In 15 participants, the model achieved a classification accuracy of 99.06%; after continuous EEG was converted into spike sequences using delta modulation, accuracy remained at 97.56%. However, high classification performance did not directly translate into low-latency operation, with a per-sample inference time of 2.533 s on a conventional von Neumann platform. This finding highlights a hardware-level gap between the theoretical computational efficiency of SNNs and their practical real-time performance. Beyond computational efficiency and latency, efficient decoding must also maintain stable performance in the presence of complex neural information and interindividual variability. Gong et al. [159] proposed SGLNet, which uses a learnable spike encoder to convert continuous EEG into task-relevant spike sequences and combines spiking adaptive graph convolution with LSTM to model interchannel spatial topology and temporal dependencies. In the 4-class motor-imagery task from PhysioNet, the model achieved a mean accuracy of 84.15 ± 0.58% under mixed-subject conditions and showed a degree of generalization under cross-subject conditions. However, the original study also reported that all models performed worse under cross-subject than mixed-subject conditions, and SGLNet did not incorporate a dedicated cross-subject compensation mechanism for interindividual variability. Thus, greater computational efficiency does not automatically translate into stable real-time decoding, and practical BCIs require coordinated optimization of computational efficiency, response latency, and cross-subject reliability.

Overall, BCI decoding is affected by neural signal complexity, limited data availability, interindividual and intersession variability, and real-time computational demands, which constrain the reliable transformation of neural information into stable motor-intent outputs. To address these challenges, AI enhances the decoding capability and stability of complex neural signals through complementary strategies involving data-efficient learning, spatiotemporal and relational representation, adaptation to distribution shifts, and efficient real-time decoding. The value of AI within the information reliability chain therefore extends beyond improving decoding accuracy to maintaining the stable transfer of neural information from signal representation to motor-intent output. Further translating reliable decoding results into accurate and timely control and feedback is critical for BCI-enabled motor assistance and functional recovery.

## AI-Enabled BCIs for Motor Assistance and Rehabilitation

Following stable neural signal acquisition and reliable intention decoding, BCIs must further translate decoding outcomes into accurate motor execution and effective feedback to complete the transmission of the information reliability chain from neural representation to functional output [160]. For motor assistance and rehabilitation, system performance should therefore be evaluated not only by decoding accuracy but also by outcomes at different levels, including control stability, feedback timing, and functional improvement. This section focuses on AI-enabled BCI applications in motor assistance and rehabilitation, examining how reliable neural information is translated into motor functional outputs and rehabilitation benefits through external effectors and closed-loop feedback.

### AI-enabled BCIs for motor assistance

In motor assistance, BCIs translate AI-decoded neural intentions into control commands for external effectors, thereby bypassing impaired neuromuscular pathways to enable intention-driven motor output [161]. Within the information reliability chain, the key at this stage is to translate reliable decoding into accurate, timely, and stable control, requiring consideration of control accuracy, response latency, and continuous execution stability. The following section discusses different motor assistance approaches and their control performance.

#### BCI-driven exoskeletons

Exoskeletons establish mechanical coupling with human limbs to provide assistive torque, postural support, and guided movement trajectories for impaired or weakened extremities [162,163]. In BCI-based motor assistance, the primary value of exoskeletons extends beyond simply substituting for lost motor function; instead, they enable the real-time transformation of neural activity generated during MI or motor attempts into perceivable motor feedback. Following the generation of motor intention, changes in sensorimotor rhythms recorded by EEG—particularly event-related desynchronization (ERD) within the μ and β bands—can be decoded and translated into corresponding exoskeleton-assisted movements [164]. When neural intention, mechanical execution, and sensory feedback are temporally aligned within a closed-loop framework, repetitive training may reinforce motor-related cortical activity and facilitate activity-dependent neuroplasticity, ultimately supporting functional recovery [165,166]. Accordingly, BCI–exoskeleton performance should not be evaluated solely by decoding accuracy but should consider intention recognition accuracy, response latency, false-trigger rate, continuous control stability, and feedback timing according to the specific rehabilitation task. The priorities of these parameters vary across motor tasks and should be balanced according to movement type, control complexity, and rehabilitation goals. Upper-limb BCI–exoskeleton systems primarily target the restoration of wrist, finger, and forearm motor function, with performance evaluation emphasizing motor intention recognition accuracy, control degrees of freedom (DOFs), and system wearability. For example, Wu [167] developed a lightweight cognitively driven forearm exoskeleton that integrated low-cost EEG acquisition, the BrainFormer deep-learning model, and a forearm actuator with 5 DOFs into a closed-loop system with real-time vibrotactile feedback (Fig. 5A, I to III). The system achieved over 92.3% accuracy in recognizing brain-intention commands and 100% accuracy in classifying Alpha/Beta-wave suppression states, while maintaining an exoskeleton weight below 1.5 kg and enabling multi-DOF movements of the elbow, wrist, and palm. Under operating load, the maximum structural displacement was only 0.0027 mm, indicating good mechanical stability. However, the study primarily validated predefined movement control based on steady-state visual evoked potential (SSVEP) and binary brain-state classification in a limited sample under controlled experimental conditions. The high classification accuracy therefore mainly reflects technical feasibility for discrete commands and does not yet establish the system’s ability to adapt to dynamic intention transitions and real-time state switching during continuous movement.

**Fig. 5.**
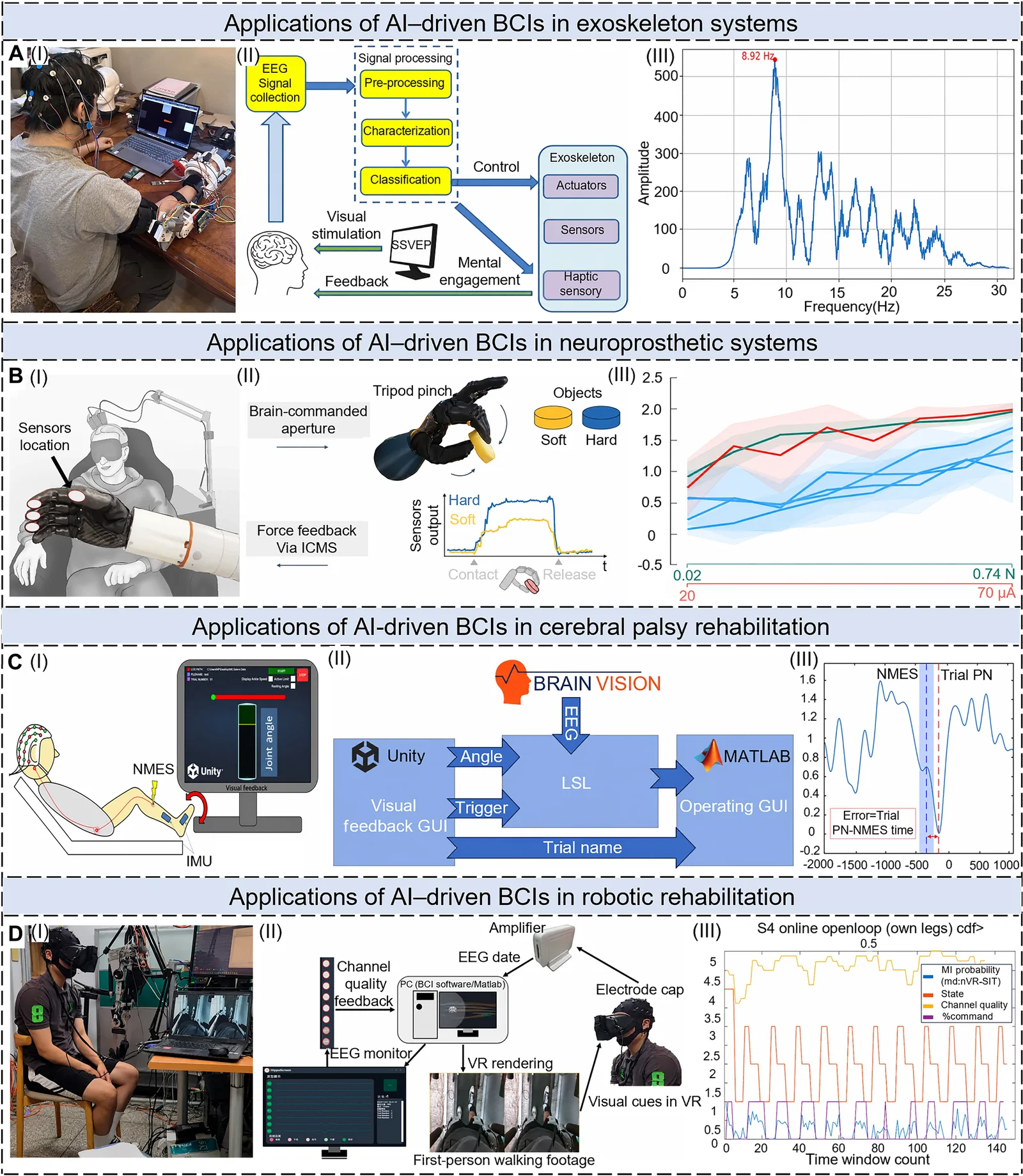
AI-enabled BCIs for motor assistance and rehabilitation. (A) Applications of AI-driven BCIs in exoskeleton systems: (I) photograph of a participant using the wearable EEG–exoskeleton system; (II) workflow of the AI-based decoding framework for wearable EEG-driven exoskeleton control; (III) frequency-domain characteristics of BCI signals. Reproduced from Ref. [167] under the CC BY-NC-ND 4.0 license. Copyright © 2025 The Author. (B) Applications of AI-driven BCIs in neuroprosthetic systems: (I) experimental task paradigm designed to simulate real-world environments; (II) brain-controlled prosthetic grasping and the mechanism of intracortical microstimulation-based force feedback; (III) comparison of tactile perception under different stimulation strategies, including single-electrode stimulation, multi-electrode stimulation, and biomimetic mechanical feedback. Reproduced from Ref. [177] under the CC BY-NC-ND 4.0 license. Copyright © 2024 The Author(s). (C) Applications of AI-driven BCIs in cerebral palsy rehabilitation: (I) structural design integrating MI-BCI with neuromuscular electrical stimulation (NMES); (II) software data flow and synchronization framework of the BCI-NFT system; (III) temporal difference between trial-specific MRCP peak negativity and NMES activation. Reproduced from Ref. [185] under the CC BY 4.0 license. Copyright © 2024 Behboodi, Kline, Gravunder, Phillips, Parker, and Damiano. (D) Applications of AI-driven BCIs in robotic rehabilitation systems: (I) experimental scenario under virtual reality (VR)-enhanced MI-BCI conditions; (II) structural design of a VR-enhanced MI-BCI system; (III) online MI probability and system-state outputs during open-loop testing. Reproduced from Ref. [197] under the CC BY 4.0 license. Copyright © 2024 The Author(s).

Lower-limb exoskeletons impose greater demands on BCI decoding. Unlike discrete upper-limb tasks such as grasping or flexion–extension, gait control requires the identification of initiation, maintenance, and termination intentions during continuous movement. Performance evaluation should therefore further consider start/stop accuracy, response time, false triggers, and continuous control stability. Ferrero et al. [168] proposed an asynchronous deep-learning EEG–BCI framework for gait control, using separate static and dynamic models to identify gait initiation and termination intentions and continuous EEG windows to enable state transitions. After full-layer fine-tuning, mean classification accuracies under static and movement conditions were 64.1% and 60.1%, respectively, indicating reduced decoding performance during movement. In closed-loop experiments, the static model showed relatively high initiation reliability, whereas stop control with the dynamic model was less stable, with the ratio of stops remaining below 65%. For lower-limb BCI–exoskeleton systems, offline classification accuracy alone is therefore insufficient to reflect actual control performance; task-specific measures such as start/stop success rates, response time, and false-trigger rate provide a more direct assessment of reliability during continuous gait control. However, even when stable control is achieved in individual participants, interpatient differences in neural representations may limit model transfer to broader rehabilitation populations.

Accordingly, the role of exoskeletons is gradually extending from immediate motor assistance toward modulation of motor-related neural responses. In post-stroke motor rehabilitation, interpatient variability in neural activity can limit model transfer, making neural response consistency and cross-subject generalization important evaluation parameters in addition to decoding accuracy. Zhang et al. [169] used an exoskeleton to guide standardized passive hand movements, improving the consistency of neural responses through stable proprioceptive input. Event-related potential (ERP) amplitudes over the contralateral and ipsilateral motor cortices in healthy participants reached −7.29 ± 4.51 μV and −4.33 ± 3.69 μV, respectively, with cortical potentials rapidly returning to baseline after passive movement. In cross-subject decoding, EEGNet achieved classification accuracies of 86.00% and 72.63% for healthy participants and patients with stroke, respectively, compared with 64.75% and 67.81% under voluntary movement conditions. For practical clinical application, BCI–exoskeleton systems must further consider portability, wireless operation, operational efficiency, and adaptability to functional tasks. Colucci et al. [170] developed a fully portable hybrid brain/neural exoskeleton system that used wireless EEG/electrooculography (EOG) to detect sensorimotor rhythm (SMR)-ERD and eye-movement signals in real time, controlling a wearable hand exoskeleton with a processing latency of <100 ms during self-paced bimanual activities of daily living. In 5 patients with stroke, mean EEG control accuracy reached 83%, with a mean SMR-ERD initiation time of 1.65 s. All patients completed 10 bimanual daily-living tasks during their first training session, while system setup and calibration required <20 min. However, this study involved only a small-sample, single-session feasibility assessment and did not evaluate motor functional improvement after long-term training. Overall, performance parameters for BCI–exoskeleton systems should be selected according to specific rehabilitation tasks and jointly evaluated along the information reliability chain of “neural signal acquisition–intention decoding–mechanical execution–sensory feedback”, thereby providing an integrated assessment of the reliability of neural intention decoding, device control, and closed-loop feedback.

#### BCI-driven neuroprostheses

BCI-driven neuroprostheses acquire central neural activity through implanted or high-resolution neural interfaces and decode these signals into control commands for external prosthetic devices, speech synthesizers, or stimulation-based feedback systems [171,172]. Unlike exoskeletons, which primarily assist movement using preserved limbs, neuroprosthetic systems place greater emphasis on the substitution and restoration of impaired motor, sensory, or communication functions. Their fundamental principle lies in establishing bidirectional neural communication. On one hand, intention-related information is decoded from motor- or language-associated cortical activity to drive external effectors; on the other hand, sensory information—including tactile sensation, pressure, or movement outcomes—is transmitted back to the nervous system through cortical stimulation or peripheral feedback, thereby forming a closed-loop control pathway integrating intention decoding, prosthetic execution, and sensory feedback [173,174]. Performance requirements vary with the targeted function: Communication restoration emphasizes decoding speed and accuracy, sensory restoration requires stimulus discriminability and perceptual stability, whereas complex motor prostheses depend more strongly on continuous control accuracy, DOFs, and real-time performance [175]. Among these applications, speech restoration represents an important direction for communication replacement. In individuals with severe paralysis or aphasia, effective output through the speech apparatus may be impaired, while language-related cortical activity can retain decodable information. Willett et al. [176] implanted microelectrode arrays in human speech-related cortical regions and combined an RNN with a language model to decode attempted speech in real time and convert it into text. The system achieved a word error rate (WER) of 9.1% with a 50-word vocabulary and 23.8% with an expanded 125,000-word vocabulary, while reaching a real-time decoding rate of 62 words min^−1^, 3.4-fold higher than the previous record for BCI communication speed. However, increasing vocabulary size also increased decoding difficulty, as reflected by the rise in WER from 9.1% to 23.8%. This result indicates that neuroprostheses for open-ended daily communication require further trade-offs among vocabulary coverage, decoding accuracy, and communication efficiency.

As neuroprostheses expand from communication replacement to motor function restoration, performance requirements also shift from unidirectional information output toward bidirectional neural interaction. Sensory feedback becomes critical for fine motor control, extending evaluation to perceptual stability, tactile localization, and force-intensity discrimination. Greenspon et al. [177] used multi-electrode intracortical microstimulation to evoke stable and spatially localized artificial tactile sensations and improve closed-loop prosthetic-hand grasping (Fig. 5B). Multi-electrode arrays covering the hand somatotopic region provided spatially differentiated tactile encoding, allowing sensor signals from the prosthetic hand to be converted into real-time cortical stimulation during grasping. When objects of different softness were contacted, force-sensor signals were converted into stimulation parameters, enabling discrimination of pressure changes and differences in softness. Multi-electrode stimulation also markedly improved tactile localization compared with single-electrode stimulation: 30% of single-electrode trials failed to evoke localizable sensations, and 42% of perceptible trials were incorrectly localized, whereas multi-electrode stimulation reduced these rates to 0% and 7%, respectively (Fig. 5B, I to III). However, artificial sensory feedback remains substantially less precise than natural touch. Within the safe stimulation range, single-electrode stimulation provided only 2 to 14 discriminable intensity levels, with a median of 7, compared with approximately 45 to 50 for natural touch. Thus, although current neuroprostheses can provide basic location and force feedback, finer grasping requires further improvements in sensory resolution and dynamic range. Building on this approach, Valle et al. [178] used spatiotemporally patterned multi-electrode intracortical microstimulation to encode higher-dimensional tactile features, including edges and motion. Participants identified 4 motion directions with accuracies of 76 ± 14% and 78 ± 15%, while the Weber fraction for motion-speed discrimination was 0.56 ± 0.09, approaching the range of natural touch. Artificial sensation was thereby extended from basic location and intensity feedback to the encoding of motion direction and speed, expanding the dimensionality of tactile information available to neuroprostheses.

Motor control of neuroprostheses is also progressing from discrete movement triggering toward high-degree-of-freedom posture reconstruction, making continuous trajectory accuracy, postural stability, and multi-DOF control increasingly important. Agudelo-Toro et al. [179] recorded hand posture-related neural activity from the anterior intraparietal area, F5, and M1 regions of nonhuman primates and used joint posture–velocity decoding to control a multi-DOF virtual prosthetic hand in real time during power and precision grips. Compared with the Kalman filter alone, FTTraj/NARX substantially reduced posture-holding variance, trajectory redundancy, and trajectory error while improving overall task success, bringing BCI-controlled grasp trajectories and maintained postures closer to natural hand movements. Posture decoding performance further improved with training, accompanied by increased separation and dimensionality of neural representations, indicating that changes in neural representation occurred alongside improved high-DOF decoding performance. However, control of untrained postures remained limited by the coverage of training trajectories, indicating that improved performance under trained conditions does not fully represent generalization to novel postures and natural movements. Cross-posture generalization therefore remains a key limitation as high-DOF neuroprostheses progress from predefined tasks toward more naturalistic operation.

### AI-enabled BCIs for motor rehabilitation

Building on intention-driven motor output achieved through motor assistance, BCI-based motor rehabilitation further focuses on improving patients’ own motor function. By forming a closed loop among neural intention, motor execution, and sensory feedback, repeated training can modulate motor-related neural activity and support neuroplasticity and functional recovery [180]. Within the information reliability chain, the focus of evaluation therefore extends beyond device control to neurophysiological changes and clinical functional benefits. The characteristics and functional outcomes of BCI rehabilitation interventions across different patient populations are summarized in Table S2.

#### Application of BCIs in CP rehabilitation

CP differs from acquired adult conditions such as stroke and SCI, as its primary pathological basis involves developmental central nervous system injury leading to impaired motor control, postural dysfunction, and disrupted sensorimotor integration [181]. Accordingly, CP rehabilitation focuses not only on compensating for motor deficits but also on leveraging neuroplasticity in children to promote reorganization of abnormal motor networks and optimization of motor control patterns. Given the limited tolerance of children for invasive devices, prolonged training, and high cognitive demands, BCI-based CP rehabilitation is better suited to noninvasive neural recording, low-burden decoding, and gamified or robot-assisted feedback, integrating motor intention training, peripheral sensory input, and cognitive modulation into sustainable rehabilitation protocols [182]. Accordingly, performance evaluation should extend beyond decoding accuracy to consider child-specific requirements, including neuromodulatory effects, feedback timing, cognitive load, and long-term adherence [183]. The integration of BCIs with robotic training provides an active rehabilitation approach for children with CP. Qi et al. [184] developed a closed-loop MI-BCI–lower-limb robotic system in which MI drove robotic movement and the resulting proprioceptive input was fed back to the central nervous system. Compared with the resting state, the duration of microstate D, associated with attention and motor execution, increased from 77.02 to 86.79 ms under the MI-BCI condition, while its coverage increased from 23.84% to 28.70%; functional brain connectivity was also enhanced across multiple frequency bands. These neurophysiological changes indicate that, for CP rehabilitation aimed at motor-network reorganization, cortical activity and functional connectivity can complement decoding performance in characterizing neural responses to BCI–robotic training. However, robotic feedback primarily relies on movement-induced proprioceptive input, leaving relatively limited direct temporal coupling between central motor intention and target muscle activation.

To strengthen the temporal coupling between central intention and peripheral muscle activation, BCIs can be combined with neuromuscular electrical stimulation (NMES). Behboodi et al. [185] developed an movement-related cortical potential (MRCP)-based BCI-NFT system in which an LSTM detected ankle dorsiflexion intention in real time and triggered NMES of the tibialis anterior, temporally aligning sensory feedback with motor-related cortical activation (Fig. 5C, I to III). The system detected motor intention within an MRCP window approximately 500 ms before movement onset. Cross-session transfer learning reduced the number of calibration trials from 50 to 25 while maintaining a detection accuracy of 80.8%, although the false-trigger rate remained approximately 17%. The participant completed 100 movement attempts in each of 10 training sessions, and response delays observed during the initial session were corrected and remained well controlled in subsequent sessions. However, the study included only one participant with CP and primarily established the technical feasibility of the closed-loop system, precluding conclusions regarding generalizability or long-term motor recovery. Beyond technical feasibility, subsequent studies have examined whether BCI training translates into functional motor improvement. Bobrov et al. [186] applied an MI-BCI to CP rehabilitation, providing matched proprioceptive feedback following real-time recognition of motor intention. During training, MI-induced μ-rhythm desynchronization was significantly correlated with BCI control accuracy. After 7 to 10 d of training, the total Fugl–Meyer active-movement score increased by 7 points and the total action research arm test (ARAT) score by 7.5 points, suggesting that closed-loop BCI training not only can provide intention-driven feedback but also may promote upper-limb motor recovery. In addition to functional improvement, the effects of sustained BCI training on motor-related neural activity have also been investigated. Xie et al. [187] used repeated MI-BCI rehabilitation training in children with CP, with real-time feedback guiding voluntary modulation of MI-related brain activity. After up to 60 d of training, relative power in the 8- to 15-Hz band during left/right MI increased from 0.66/0.70 to 0.84/0.83, accompanied by an increased brain-network clustering coefficient and reduced characteristic path length, reflecting improved efficiency of motor-related neural information processing following sustained MI-BCI training. However, standardized clinical motor outcomes were not concurrently evaluated. Overall, BCIs provide a promising rehabilitation approach for CP by strengthening closed-loop coupling among motor intention, sensory feedback, and neural activity, thereby supporting active motor training and neural modulation. Nevertheless, long-term motor functional benefits require further validation in larger cohorts and longer-term studies.

#### BCI-driven rehabilitation robots

Rehabilitation robots serve as key execution platforms within closed-loop BCI-based rehabilitation systems, offering advantages including highly repetitive training, adjustable intensity, quantifiable therapeutic protocols, and simultaneous acquisition of kinematic and behavioral parameters [188,189]. Within the information reliability chain, rehabilitation robots function not only as movement execution modules but also as interfaces that translate decoded motor intentions into standardized training tasks while promoting active patient engagement through visual, proprioceptive, and tactile feedback [190]. Unlike conventional mechanically assisted rehabilitation, BCI-driven robotic rehabilitation emphasizes temporal coupling among neural intention, motor execution, and feedback regulation, thereby shifting the rehabilitation process from passive mobilization toward active neuromodulation [191]. Accordingly, their performance should be evaluated not only by intention recognition accuracy but also by task completion, clinical functional improvement, neurophysiological changes, and closed-loop feedback stability according to specific rehabilitation goals. Upper-limb rehabilitation robots have provided initial evidence for the feasibility of BCI-based active motor training. Kim et al. [192] integrated EEG MI and EOG signals to control a robotic hand, assisting patients with stroke in grasping and bimanual activities of daily living, with functional performance assessed using the 10-task BeBiTS. Of 8 patients, 4 adapted to BCI training and completed the robot-assisted assessment, with greater assistance benefits observed for tasks with baseline BeBiTS item scores ≤4. These findings demonstrate the feasibility of using brain signals as an active control source to shift robotic training from predefined trajectory execution toward patient intention-driven assistance. However, the fact that only a subset of patients adapted to BCI training also indicates that performance may depend on individual neural status and BCI-use capability, and its applicability to broader stroke populations requires further validation. Cantillo-Negrete et al. [193] further evaluated the clinical rehabilitation effects of ReHand-BCI in a randomized controlled trial. After 30 sessions of MI-driven robotic hand training, the median Fugl-Meyer assessment of the upper extremity (FMA-UE) score in the experimental group increased from 24.5 to 28, while the ARAT score increased from 8.5 to 20, accompanied by trends toward enhanced ipsilesional cortical activity and corticospinal tract integrity. These findings provide preliminary evidence for potential clinical and neurophysiological effects of BCI–robotic training. However, neither the clinical nor neuroplasticity-related outcomes showed significant between-group differences, and the relationship between closed-loop BCI engagement, cortical plasticity, and functional recovery requires more direct multimodal evidence. To further examine the relationship between functional improvement and changes in neural activity, Ji et al. [28] used an EEG-BCI to control a soft robotic glove and compared it with identical robotic training without BCI control in a randomized controlled design. After 4 weeks of training, ARAT and Fugl-Meyer assessment of the upper limb (FMA-UL) scores improved by 14 and 18 points in the BCI group, respectively, compared with 7 and 7 points in the control group (*P* = 0.032 and 0.010). fNIRS further revealed significantly increased activation of the bilateral sensorimotor cortices and medial prefrontal cortex in the BCI group, whereas no significant changes were observed in the control group. Moreover, changes in prefrontal cortical activation were significantly positively correlated with ARAT improvement (*r* up to 0.671), further indicating an association between BCI closed-loop training-related neural activity changes and improvements in upper-limb function.

In lower-limb rehabilitation, BCI–robotic systems have gradually expanded from motor assistance toward gait-rhythm modulation and neurofeedback training [194]. Serafini et al. [195] combined MI-BCI with Lokomat-assisted gait training, using multichannel EEG neurofeedback to guide patients with SCI in modulating μ/β sensorimotor rhythms. After 24 sessions over 12 weeks, 6 of 7 patients achieved clinically important improvements in neurogenic bladder symptom score (NBSS), accompanied by significant improvements in trunk balance. Further analysis revealed significant correlations between μ/β rhythm modulation and multiple NBSS domains (|*r*| up to 0.93). Building on these findings, enhancing voluntary regulation of motor-related rhythms while maintaining stable neural representations has become important for improving the reliability of closed-loop BCI rehabilitation. Blanco-Diaz et al. [196] incorporated visual neurofeedback into MI-BCI–Lokomat gait training, providing real-time feedback of μ/β rhythms to guide patients with SCI in voluntarily modulating motor cortical activity. The system achieved a mean classification accuracy of 74.4% and a visual neurofeedback performance of 76.3%. After training, the clustering variance of MI features decreased, while class separability increased, suggesting that visual neurofeedback may improve the stability of MI-related neural representations. However, the feedback remained primarily based on visual presentation of neural rhythms, providing limited support for immersive motor perception and continuous closed-loop interaction during actual gait.

To strengthen the coupling among motor intention, sensory feedback, and actual gait execution, Lin and Sie [197] integrated virtual reality (VR)-enhanced MI-BCI with a lower-limb exoskeleton. A first-person walking scenario was used to reinforce MI, while EEG decoding outputs triggered exoskeleton-assisted gait training. The system further employed cumulative distribution function (CDF)-based adaptive threshold calibration to transfer the BCI model trained under static conditions to actual exoskeleton control. The experimental setup, system architecture, and representative online outputs during open-loop testing are shown together (Fig. 5D, I to III). Open-loop testing achieved an accuracy of 75.35%, while the correct triggering rate for rehabilitation movements during closed-loop operation reached 74%. Compared with visual neurofeedback alone, this system established a more direct closed-loop pathway of “motor intention–BCI decoding–exoskeleton execution–multisensory feedback”, coupling neural intention decoding with actual gait execution and multisensory feedback. Current evidence indicates that BCI–rehabilitation robots are progressing from technical feasibility demonstrations toward evaluations of clinical function and neurophysiological effects. Upper-limb studies place greater emphasis on functional improvement and associated changes in neural activity, whereas lower-limb studies focus more on motor intention recognition, neural-rhythm modulation, and closed-loop triggering stability. However, interpatient variability in BCI adaptability, the relationship between neural changes and functional recovery, and the effects of long-term training require further validation.

Overall, AI-enabled BCIs can translate neural intentions into external device control and establish closed-loop interactions through motor execution and sensory feedback, providing an important technical basis for motor assistance and rehabilitation. However, from the downstream perspective of the information reliability chain, technical success does not necessarily translate into sustained neuroplasticity or long-term functional recovery. Although current studies have reported improvements in control performance, neurophysiological measures, and clinical function, part of the evidence remains derived from small cohorts, short intervention periods, or selected patient populations, with substantial heterogeneity in intervention protocols and outcome assessments. Evaluation of the rehabilitative value of BCIs should therefore extend beyond immediate decoding and device control to changes in neural function, clinical functional improvement, and long-term retention, thereby determining whether reliable information transmission ultimately translates into sustained rehabilitation benefits.

## Conclusion and Perspectives

BCIs are evolving from simple neural signal transmission channels into neurorehabilitation systems that integrate neural interfaces, intelligent decoding, control execution, closed-loop feedback, and functional recovery. This review follows the progression “from neural interfaces to functional recovery” and examines the information reliability chain in motor rehabilitation BCIs. Different neural recording modalities constitute the starting point of neural information acquisition, while the biocompatibility, electrical and electrochemical properties, interfacial adhesion, mechanical matching, and fatigue reliability of neural interface materials further determine material–tissue coupling and long-term signal stability. AI subsequently represents and decodes dynamic, nonstationary neural information, which is translated through external effectors and closed-loop feedback into motor outputs and rehabilitation training. Thus, the long-term reliability of BCIs is not determined by a single material property, instantaneous decoding accuracy, or a specific type of end device, but by whether neural information can be transmitted continuously and reliably along the information reliability chain. In this process, neural signals are not static inputs but dynamic information streams that continuously vary with interface conditions, physiological environments, and training. Accordingly, the role of AI should extend beyond instantaneous motor intention recognition to adaptation to cross-session drift, interindividual variability, and changes in task states. External effectors further recouple neural intentions with motor outcomes through multimodal feedback, providing closed-loop training conditions for neuroplasticity modulation and functional recovery.

Despite substantial advances in neural interface materials, AI-based decoding, and closed-loop BCIs, long-term reliability and clinical translation remain constrained by multilevel challenges across the information reliability chain. First, long-term neural interface stability remains a fundamental bottleneck in upstream information reliability. In invasive BCIs, chronic tissue responses, glial encapsulation, mechanical mismatch, degradation of electrochemical performance, and loss of functional recording channels can progressively compromise interface and signal stability. In noninvasive BCIs, sweat, hair, skin conditions, motion artifacts, and wearing stability continue to limit long-term recording quality under real-world conditions. Second, current AI decoding models still rely heavily on offline training and subject-specific calibration and remain limited in adapting to cross-session, cross-task, and cross-subject neural variability. Generalization with limited data, online updating, and uncertainty estimation therefore require further improvement. Moreover, improved decoding performance does not necessarily translate into stable control and feedback. Response latency, false triggering, and variations in feedback timing and modality can disrupt closed-loop information transmission, while heterogeneity in task paradigms and feedback strategies limits comparisons across studies. Finally, current clinical evidence remains largely constrained by small cohorts, short intervention periods, and selected patient populations. Long-term follow-up, multicenter validation, and standardized outcome assessment remain insufficient, and whether technical improvements ultimately translate into sustained neurophysiological changes and long-term functional benefits has yet to be adequately established.

Future motor rehabilitation BCIs should therefore progress from local performance optimization toward cross-level coordinated design centered on the information reliability chain. Neural interface materials should first be optimized across multiple properties to support long-term material–tissue coupling and signal stability while establishing dynamic relationships between interface states and signal quality. On this basis, AI should further identify and adapt to upstream interface and signal changes through cross-session and cross-subject adaptation, online updating, and uncertainty estimation, thereby limiting the downstream propagation of reliability loss. Control and feedback systems should further adjust their outputs according to decoding reliability, allowing downstream compensation for upstream reliability variations and progressively establishing a cross-level closed loop of “interface-state sensing–signal-quality assessment–reliable decoding–adaptive control and feedback”. Ultimately, clinical studies should establish longitudinal relationships among interface stability, decoding and control performance, and functional outcomes to determine whether technical optimization translates into sustained rehabilitation benefits. Along this trajectory, next-generation BCIs may evolve toward long-term personalized closed-loop systems in which reliability can be monitored, failures can be traced, and downstream compensation can be implemented.

## Supplementary Materials

Supplementary 1 Fig. S1 Tables S1 and S2 References [203–209]
