# Supplementary material for: Engineering Neural Interface Materials for Brain–Computer Interfaces: Toward Reliable Neural Information and Functional Recovery
Source: Biomater Res. 2026 Nov 3;2026:0437. doi: 10.34133/bmr.0437 (PMC13728234; doi:10.34133/bmr.0437)
Supplement: Supplementary file 1 [file bmr.0437.f1.docx]

**Figure S1. Classification and signal acquisition mechanisms of BCIs.** **A–C. Non-invasive BCIs:** **A.** Schematic illustration of the neural sources of non-invasive EEG signals and the associated volume conduction mechanisms. Reproduced from Ref. [56] under the CC BY-NC-ND 4.0 license. Copyright © 2020 Epileptic Disorders. **B.** Schematic illustration of signal acquisition using non-invasive fNIRS and the underlying neurovascular coupling mechanisms. Reproduced from Ref. [60] under the CC BY 4.0 license. Copyright © 2018 The Author(s). **C.** Schematic illustration of the signal sources and imaging mechanisms of non-invasive MEG. Reproduced from Ref. [63] under the CC BY-NC-ND 4.0 license. Copyright © 2024 The Author(s). **D–F. Semi-invasive BCIs:** **D.** Schematic illustration of semi-invasive ECoG electrodes and array configurations. Reproduced from Ref. [68] under the CC BY 4.0 license. Copyright © 2023 The Author(s). **E.** Comparative schematic of semi-invasive ECoG in terms of spatial resolution and application domains. Reproduced from Ref. [70] under the CC BY 4.0 license. Copyright © 2024 The Author(s). **F.** Clinical implantation schematic of high-density semi-invasive ECoG for recording cortical language activity. Reproduced from Ref. [74] with permission from Elsevier. Copyright © 2011 Elsevier Inc. **G–H. Invasive BCIs:** **G.** Schematic illustration of invasive sEEG with multi-trajectory depth electrode implantation. Reproduced from Ref. [78] under the CC BY-NC-ND 4.0 license. Copyright © 2017 The Japan Neurosurgical Society. **H.** Implantation and localization workflow of invasive sEEG for functional mapping of motor and language areas. Reproduced from Ref. [79] under the CC BY-NC-ND 4.0 license. Copyright © 2025 The Author(s).

**Table S1. Performance, advantages, and limitations of AI-enabled BCIs.**

| Reliability Challenges | | AI model | BCI type | Application | Performance | Advantages | Limitations | Refs. |
| --- | --- | --- | --- | --- | --- | --- | --- | --- |
|  | Limited neural data | SVM | EEG | Four-class MI classification (left hand, right hand, feet, rest). | Accuracy: 78.82%; F1-score: 77.0%. | Suitable for high-dimensional EEG feature classification; good classification performance. | Reliance on handcrafted features and preprocessing; lack of online closed-loop validation. | [123] |
|  |  |  | EEG + fNIRS | Left/right-hand MI classification. | Accuracy: 88.33 ± 5.80%. | Multimodal feature fusion significantly improves MI decoding accuracy and reduces redundant features. | Only 18 healthy participants; binary classification and offline validation; lack of online closed-loop control validation. | [203] |
|  |  | DBN | EEG | Four-class MI classification (left hand, right hand, feet, tongue). | Accuracy: 86.14%. | SSA optimizes DBN hyperparameters, improving MI decoding accuracy and stability. | Self-collected data from only 8 participants; lack of online closed-loop control validation. | [128] |
|  |  |  | fMRI | Seven-class cognitive/motor task decoding. | Accuracy: 97.86 ± 3.42%. | Extracts multi-layer spatiotemporal features from small-sample fMRI data and improves feature interpretability. | Offline fMRI decoding;  no online closed-loop control. | [204] |
|  |  | GAN | EEG | MI classification (left hand, right hand, both feet, tongue). | Accuracy: 81.3%; Kappa: 0.751. | Generates synthetic EEG data to augment training data, alleviating small-sample limitations and improving decoding accuracy and stability. | Validated on two public datasets, without online closed-loop evaluation. | [131] |
|  |  |  | EEG | Cross-subject left/right-hand MI classification. | RMSE: 0.21;  FD: 0.75;  DTW: 10.38. | Learns EEG temporal distributions and generates realistic synthetic data, alleviating data scarcity and improving downstream performance. | Generated-data quality depends on adversarial training; validated on only one public dataset. | [132] |
|  | Spatio- temporal/  relational represen- tation | CNN | EEG | Four-class MI classification (left fist, right fist, both fists,  both feet). | Accuracy: 96.59%. | Automatically learns high-level EEG features, enabling high-accuracy multiclass MI decoding. | Model performance depends on preprocessing and feature extraction; validated in only 10 participants. | [205] |
|  |  |  | EEG | Finger MI classification (thumb, index finger, little finger) and real-time robotic-arm control. | Binary accuracy: 80.56%; three-class accuracy: 60.61%. | Automatically learns movement-related EEG features; fine-tuning improves cross-session decoding performance; enables real-time robotic-arm control. | Marked performance degradation in three-class classification; limited by SNR and EEG spatial resolution. | [8] |
|  |  | RNN | Invasive BCI | Bimanual motor-intention decoding and real-time dual-cursor control. | Offline decoding: right, r = 0.90;  left, r = 0.83. | Captures temporal dependencies and nonlinear relationships in neural activity; supports continuous, multi-effector motor decoding. | Prone to overfitting the temporal structure of offline data; limited cross-session generalization. | [139] |
|  |  | LSTM | EEG | Left/right-hand MI classification. | Accuracy: 83.3%; Kappa: 0.666. | Bidirectionally captures temporal dependencies in EEG, improving MI temporal feature representation and classification performance. | Relies on WST feature extraction; affected by inter-individual variability. | [206] |
|  |  | GNN | EEG | Four-class MI classification (left hand, right hand, feet, tongue). | Accuracy: 80.82%; F1-score: 82.8%. | Models non-Euclidean electrode topology and dynamic connectivity, enhancing representation of cross-channel relationships. | Relies on brain connectivity construction; risk of spurious connections. | [145] |
|  |  |  | EEG | Left/right-hand MI classification. | Accuracy: 95.82 ± 0.86%; F1-score: 94.75 ± 0.62%. | Integrates multi-domain features and dynamic cross-frequency connectivity to improve cross-subject MI decoding. | Multi-feature fusion increases model complexity and training time; performance decreases across subjects. | [146] |
|  | Distribution shift / adaptation | Transformer | EEG | Four-class/two-class MI classification. | New subjects: accuracy, 74.48%; existing subjects: accuracy, 76.10%. | Multi-head self-attention captures global temporal dependencies in EEG, improving cross-subject MI decoding. | Long-sequence computation has high memory overhead; 3.5-s signal windows result in relatively high latency. | [150] |
|  |  |  | EEG | four-class/two-class MI | Four-class: accuracy, 82.95%,  κ = 0.7726;  two-class: accuracy, 88.00%, κ = 0.7599. | Models global dependencies among multi-scale EEG features, improving MI decoding performance. | Increased model parameters and computational overhead; lack of real-time deployment and cross-subject generalization validation. | [207] |
|  | Real-time closed-loop operation | SNN | EEG | Four-class MI classification (left hand, right hand, both feet, tongue). | Accuracy: 68.92%; cross-subject: 52.13%. | Sparse spike-based event-driven computation, enabling spatiotemporal EEG representation with low power consumption. | Validated only on public datasets; high-channel-count EEG may increase computational load. | [157] |
|  |  |  | EEG | Four-class MI classification (left fist, right fist, both fists,  both feet). | Accuracy:  84.15 ± 0.58%; | Spike-based encoding integrates spatial topology and temporal dependencies, improving spatiotemporal MI-EEG decoding. | Marked performance degradation across subjects; not yet validated in real-world neurorehabilitation or BCI applications. | [159] |

**Table S2. Applications and key outcomes of BCIs in motor rehabilitation.**

| BCI intervention | Patient population | Sample size | Intervention protocol | Comparator/  control group | Key outcomes | Follow-up period | Refs. |
| --- | --- | --- | --- | --- | --- | --- | --- |
| MI-BCI + rehabilitation robot | Children with spastic diplegic CP,4–6 years, GMFCS II–III. | 20 | Single-session experiment;  MI-BCI, 10 min. | Within-subject comparison. | Significant changes in resting-state EEG and functional connectivity. | NR | [184] |
| LSTM-based EEG-BCI | Child with unilateral CP, GMFCS I. | 1 | 10 sessions. | Self-controlled  (no separate control group). | Maximum ankle dorsiflexion angular velocity in sitting increased by 75.4% (133.4→234.0°/s, P = 0.001). | NR | [185] |
| EEG neuro-feedback BCI | Children with CP and  attention deficits. | 19 | 10 weeks,20 sessions. | Control: usual rehabilitation care without neurofeedback. | Decreased θ/β ratio (P = 0.04) and improved visuospatial ability (P = 0.01–0.02), indicating enhanced attention and cognition. | NR | [208] |
| MI-EEG BCI | Children with CP. | 14 | 7–10 sessions. | Self-controlled  (no separate control group). | FMA +7 points; ARAT +7.5 points; improved hand functional tasks. | NR | [186] |
| MI-based EEG-BCI | Children with CP,  8–12 years. | 18 | Short-term:12 d; long-term: 60 d; 30 min/d. | Self-controlled  (no separate control group). | Increased movement-related EEG power and brain-network efficiency, with greater long-term improvements. | NR | [187] |
| MI-based EEG-BCI | Patients with  ischemic stroke. | 296 | 1 month. | Control: traditional rehabilitation only. | FMA-UE increased by 13.17 points; ARAT increased by 14.79 points. | 3 months. | [209] |
| MI-based EEG-BCI | Patients with subacute/  chronic stroke. | 23 | 6 weeks,30 sessions. | Control: sham-BCI therapy. | FMA-UE increased from 24.5 to 28; ARAT increased from 8.5 to 20. | 6 months after baseline. | [193] |
| MI-based EEG-BCI + soft robotic glove | Patients with  subacute stroke. | 40 | 4 weeks,  20 sessions. | Control: soft robotic glove (SRG) training without BCI control. | ARAT increased from 3 to 17;  FMA-UL increased from 22 to 39.5. | NR | [28] |
| MI-based EEG-BCI + robotic gait training | Patients with chronic  complete SCI. | 7 | 12 weeks,  24 sessions. | No control group  (case series). | Significant improvement in trunk balance; 6/7 patients achieved clinically meaningful improvement in NBSS. | 30 days after intervention. | [195] |
| Gait MI-based EEG-BCI + Lokomat | Patients with complete  traumatic SCI. | 2 | 6 weeks, 12 sessions. | Within-subject comparison. | Enhanced motor-cortex rhythm modulation in most patients. | NR | [196] |
